# Transcriptome and metabolome analysis of *Atractylodes lancea* across different developmental stages

**DOI:** 10.3389/fpls.2025.1720423

**Published:** 2025-11-28

**Authors:** Guanyu Zhang, Zhiqiang Zhao, Yaqian Li, Leheng Zhang, Jimei Lu, Kangru Qi, Hua Liang, Liangping Zha, Jin Xie

**Affiliations:** 1 College of Pharmacy, Anhui Medical University, Hefei, China; 2 College of Pharmacy, Anhui University of Chinese Medicine, Hefei, China; 3 Joint Research Center for Chinese Herbal Medicine of Anhui, Anhui University of Chinese Medicine, Hefei, China; 4 MOE-Anhui Joint Collaborative Innovation Center for Quality Improvement of Anhui Genuine Chinese Medicinal Materials, Anhui University of Chinese Medicine, Hefei, China; 5 Center for Xin’an Medicine and Modernization of Traditional Chinese Medicine, Anhui University of Chinese Medicine, Hefei, China

**Keywords:** *Atractylodes lancea*, transcriptome, metabolome, terpene synthase, functional identification

## Abstract

*Atractylodes lancea* (Thunb.) DC is a medicinal plant known for its rhizome's production of valuable sesquiterpenoids, although the molecular mechanisms underlying their biosynthesis are not well understood. This study utilized integrated metabolomic and transcriptomic analyses to examine terpenoid dynamics across four developmental stages (June, July, September, November) in *A. lancea*. Metabolite profiling indicated distinct accumulation patterns: monoterpenoids reached their peak in July, while sesquiterpenoids were most abundant in September. Transcriptome analysis revealed the presence of 36 structural genes linked to the mevalonate (MVA) and methylerythritol phosphate (MEP) pathways, alongside 55 terpene synthase (TPS) genes. Subsequent phylogenetic analysis categorized the TPS genes into distinct subfamilies, and within the TPS-a subfamily, a comprehensive screening process considering significant correlations with terpenoid metabolites and the preservation of key conserved motifs identified eight candidate genes, including AlTPS21 and AlTPS42. Functional characterization demonstrated that the AlTPS21 protein catalyzes the conversion of farnesyl diphosphate to δ-cadinene and α-cadinol, while the AlTPS42 protein catalyzes the conversion of farnesyl diphosphate to δ-cadinene and α-copaene. Subcellular localization studies showed that both enzymes are localized to the nucleus and cell membrane. These findings enhance the understanding of the temporal regulation of terpenoid biosynthesis in *A. lancea and* provide crucial genetic insights for future metabolic engineering efforts.

## Introduction

*A. lancea* is a perennial medicinal plant within the genus Atractylodes, part of the Asteraceae family. The dried rhizome of *A. lancea* is a key medicinal material of traditional Chinese medicine, extensively utilized in East Asian traditional medical practices for its documented therapeutic to alleviate dampness, fortify the spleen, dispel wind and cold, and enhance vision (Jun et al., 2018). Contemporary pharmacological research has identified terpenoids found in its essential oil, such as atractylone, hinesol, β-eudesmol, and atractylodin, as the primary bioactive constituents of *A. lancea* (Xu et al., 2022). These terpenoids not only impart the characteristic aroma of *A. lancea* but are also intricately linked to a range of pharmacological activities, including anti-inflammatory (Kawada et al., 2024), antibacterial (Xu et al., 2025), antitumor (Saeheng et al., 2024; Guo et al., 2019), and regulation of gastrointestinal function (Chen et al., 2025).

Plant terpenoids are predominantly synthesized via the mevalonate (MVA) pathway in the cytoplasm and the methylerythritol phosphate (MEP) pathway in plastids (Li et al., 2023; Vranová et al., 2012), leading to the production of the essential five-carbon units, isopentenyl diphosphate (IPP) and dimethylallyl diphosphate (DMAPP). These units are subsequently converted by terpene synthases (TPSs) into a diverse array of monoterpenes, sesquiterpenes and diterpenes (Tholl, 2006; Chen et al., 2011). Despite the elucidation of these biosynthetic pathways, current research is often constrained to single time-point analyses or primarily concentrates on metabolic pathway examination (Bergman et al., 2019). The precise regulatory mechanisms that modulate these pathways throughout different developmental stages in *A. lancea*, leading to variations in metabolite accumulation, remain insufficiently characterized. Furthermore, although high-throughput sequencing has identified numerous putative TPS genes (Chen et al., 2023; Ahmed et al., 2016), the majority have not undergone experimental validation to determine their functions, substrate specificities, and roles both *in vivo* and *in vitro*, indicating a need for further investigation.

In recent years, the rapid advancement of omics technologies has significantly enhanced the application of integrated transcriptomic and metabolomic analyses in the investigation of secondary metabolite biosynthesis in medicinal plants (Yang et al., 2023; Zhao and Rhee, 2022). This integrative approach effectively correlates gene expression patterns with metabolite accumulation, offering a comprehensive perspective for elucidating biosynthetic pathways and regulatory mechanisms (Wei et al., 2025; Hua et al., 2023; Wang et al., 2023). Although prior research on *A. lancea* has predominantly concentrated on its phytochemical composition and pharmacological properties (Qiu et al., 2023; Koonrungsesomboon et al., 2014), the dynamic changes in terpenoid accumulation during rhizome development and the associated molecular regulatory networks remain inadequately explored.

This study employs an integration of metabolomic and transcriptomic analyses to examine the dynamic changes in terpenoid accumulation and gene expression in the Dabieshan variety of *A. lancea* across four developmental stages (June, July, September, November). Through differential expression analysis, KEGG enrichment, and gene-metabolite correlation analysis, key genes involved in terpenoid biosynthesis were identified. *AlTPS21* and *AlTPS42* were subsequently cloned, expressed heterologously, and functionally validated, elucidating their catalytic functions and subcellular localization. These findings provide novel insights into the developmental regulation mechanisms of terpenoid biosynthesis in *A. lancea* and lay the groundwork for varietal improvement and quality enhancement.

## Materials and methods

### Plant materials and treatment

Fresh three-year-old *A. lancea* samples were collected from Baisangguan Town, Yunyang District, Shiyan City, Hubei Province, and were authenticated by Associate Researcher Liangping Zha from Anhui University of Chinese Medicine. Rhizomes were harvested on April 20, May 20, June 18, July 19, August 20, September 20, October 20, and November 22, 2022, with three biological replicates per time point. Each sample was promptly rinsed with sterile water, and a portion was flash-frozen in liquid nitrogen and stored at –80°C for subsequent metabolomic and transcriptomic analyses. The residual material was subjected to drying at 40°C, subsequently ground, and sieved using a No. 3 mesh before being utilized for gas chromatography–mass spectrometry (GC–MS) analysis.

### GC-MS detection

Approximately 0.1 g of the powdered sample from each time point was precisely weighed and placed in a centrifuge tube for extraction with 3 mL of n-hexane via ultrasonication (40 kHz, 500 W) for 30 minutes. Following the replenishment of any weight loss, the mixture was filtered through a 0.22 μm membrane. The analysis was conducted using an Agilent 7890B–7000B GC–MS system, which was equipped with a flame ionization detector and a DB-5 MS column (60 m × 0.25 mm, 0.25 μm) under high-purity helium as the carrier gas. The GC–MS parameters were consistent with those reported in previous studies (Xu et al., 2022; Yu et al., 2019).

### Metabolomic profiling analysis

A metabolomic analysis was performed on rhizomes at four developmental stages (June, July, September, November), with three biological replicates each. Samples were ground in liquid nitrogen, mixed with saturated NaCl and an internal standard (3-Hexanone-2,2,4,4-d4), and volatile compounds were extracted using HS-SPME with a 120 μm DVB/CWR/PDMS fiber at 60°C for 15 minutes, followed by desorption at 250°C for 5 minutes. GC–MS separation used a DB-5MS column with helium at 1.2 mL/min. The temperature program was: 40°C for 3.5 minutes, increased by 10°C/min to 100°C, then 7°C/min to 180°C, and finally 25°C/min to 280°C for 5 minutes. Mass spectrometry used an EI ion source in SIM mode. Metabolites were identified with Metware’s database and analyzed with MassHunter software. PCA and OPLS-DA were used for statistical analysis, identifying differentially accumulated metabolites with VIP > 1 and FC ≥ 2 or ≤ 0.5 (Wu et al., 2023).

### RNA extraction and transcriptome analysis

The transcriptomic analysis was conducted in parallel with metabolomic studies, utilizing the same four developmental stages: June, July, September, and November. Total RNA was extracted and purified employing BGI’s Plant Total RNA Extraction Kit, with RNA integrity assessed via a Standard Sensitivity RNA Analysis Kit. Following mRNA enrichment using Oligo(dT) beads, RNA was fragmented and reverse transcribed using random hexamers to construct double-stranded cDNA libraries. These libraries underwent end-repair, A-tailing, adapter ligation, PCR amplification, and circularization, preparing them for sequencing on the MGISEQ-2000RS platform. Initial raw data were processed using SOAPnuke to yield clean reads. Functional annotation was performed with reference to the KEGG and GO databases, with a particular emphasis on terpenoid biosynthesis pathways (Ko00900, Ko00902, Ko00904, Ko00909). Differentially expressed genes (DEGs) were identified using DEGseq2, applying thresholds of |Log_2_FC| > 1 and Q ≤ 0.05. Subsequently, KEGG enrichment analysis was conducted using the Metware Cloud platform (https://cloud.metware.cn) (Liu et al., 2024b).

### Quantitative real-time PCR

RNA extracted from rhizomes at four developmental stages was reverse-transcribed into complementary DNA (cDNA) utilizing the All-in-One First-Strand Synthesis MasterMix, which includes dsDNase. Quantitative reverse transcription polymerase chain reaction (qRT-PCR) was conducted on a Bio-Rad CFX96 Touch system employing Taq SYBR^®^ Green qPCR Premix. The thermal cycling program consisted of an initial denaturation step at 95°C for 30 seconds, followed by 40 cycles of denaturation at 95°C for 15 seconds, annealing at 60°C for 30 seconds, and extension at 72°C for 15 seconds. This was succeeded by a final step at 65°C for 5 seconds and a melting curve analysis ranging from 65°C to 95°C at increments of 0.5°C every 0.5 seconds. Primers were designed using Primer Premier 5 and synthesized by General Biol (Anhui). Gene expression levels were normalized to the internal reference gene AlUBQ2 and quantified using the 2^–ΔΔCt method (Livak and Schmittgen, 2001; Arya et al., 2005). The sequences of the primers utilized are provided in **Supplementary Table S1**.

### Phylogenetic analysis

Protein sequence alignment was conducted utilizing MEGA version 11.0 software, and a phylogenetic tree was subsequently constructed employing the neighbor-joining method with 1000 bootstrap replicates. The amino acid sequences of *Artemisia annua* terpene synthases (TPSs) were sourced from the GPGenome database (http://www.gpgenome.com/species/92) as reported by Liao et al. (2022). The resulting phylogenetic tree was visualized and annotated using the Interactive Tree of Life (iTOL) tool (https://itol.embl.de/).

### Cloning of AlTPS genes

Total RNA was reverse-transcribed into complementary DNA (cDNA) using the First-Strand cDNA Synthesis Mix. Target gene sequences were amplified via polymerase chain reaction (PCR) using TransStart^®^ FastPfu DNA Polymerase with specific primers designed through Primer Premier 5.0. PCR products were separated by electrophoresis, and target bands were excised and purified using the EasyPure^®^ Quick Gel Extraction Kit. The purified products were ligated into the pEASY^®^-Blunt Zero Cloning Vector and subsequently transformed into *Escherichia coli* Trans-T1 competent cells. Positive clones were identified through colony PCR and sequenced by General Biol (Anhui). Plasmids from verified clones were extracted using the EasyPure HiPure Plasmid MiniPrep Kit and stored at –20°C for future use.

### Heterologous expression in *Saccharomyces cerevisiae* and activity assay

The *Saccharomyces cerevisiae* strain BY-T15 was employed as the host organism for conducting *in vivo* enzyme assays. The pESC-His vector, harboring AlTPS genes, along with the empty pESC-His vector, were introduced into yeast competent cells via transformation. Selection of transformants was performed on SD-His-Leu solid medium. The recombinant strains were initially cultivated in SD His-Leu dropout liquid medium supplemented with glucose at 30°C. Amplification of AlTPS genes was achieved using primers AlTPS-pESC-His-F and AlTPS-pESC-His-R for the construction of a eukaryotic expression vector. Polymerase chain reaction (PCR) was conducted to validate the recombinant strains. Subsequently, yeast cells were subjected to centrifugation, washing, and resuspension in SC-His-Leu medium containing 2.0% D-galactose to induce the expression of the target protein. Following galactose induction, the cultures were transferred to headspace vials and incubated at 30°C with agitation at 200 rpm for a duration of 2–3 days. The analysis of volatile metabolites was carried out using headspace solid-phase microextraction gas chromatography–mass spectrometry (HS-SPME-GC–MS). Extraction was conducted at 60°C for 50 min, followed by desorption at 250°C for 10 min. GC separation used a DB-5MS column (60 m × 0.25 mm × 0.25 μm) with an inlet temperature of 250°C and a split ratio of 20:1. The temperature program was: 60°C hold for 5 min, ramp at 20°C/min to 130°C, then 2°C/min to 220°C, and finally 20°C/min to 280°C hold for 5 min. Helium was used as the carrier gas at 1 mL/min. Mass spectrometry (MS) detection employed an electron ionization (EI) source with 70 eV electron energy, scanning a range of 33–600 amu. Product identification was confirmed by comparing retention times and mass spectra with authentic standards: δ-cadinene (Shanghai Yuanye Bio-Technology Co., Ltd, Cat# S22805, purity ≥95%) and α-copaene (Bio-Fount, Cat# HCC319638, purity ≥98%).

### Conserved motifs and protein structure analysis

Conserved motifs within AlTPS protein sequences were identified utilizing the MEME online tool (https://meme-suite.org/meme/tools/meme) as described by Liang et al. (2025), to facilitate the screening of candidate AlTPS genes. The results were subsequently visualized using TBtools. For multiple sequence alignment of the target proteins, AlTPS21 and AlTPS42, along with their homologs, ClustalW was employed. Homology modeling of the three-dimensional structures was performed via SWISS-MODEL (https://swissmodel.expasy.org/), using A0A5P8H4P8.1.A as a template, with visualization conducted through PyMOL.

### Subcellular localization of AlTPSs

To investigate the subcellular localization of AlTPS21 and AlTPS42 proteins, their open reading frames (ORFs) were cloned into the pBI121-GFP vector. The verified recombinant plasmids were then introduced into Agrobacterium tumefaciens GV3101 and infiltrated into the abaxial side of Nicotiana benthamiana leaves using a needleless syringe. Following an overnight incubation in darkness and a subsequent 2–3 days of standard cultivation, the epidermal layers were peeled and examined for the localization of AlTPS-GFP fusion proteins using a confocal laser scanning microscope (FV 3000, Olympus, Japan).

## Result

### Analysis of volatile products of *A. lancea* at different stages

Gas chromatography-mass spectrometry (GC-MS) analysis of *A. lancea* rhizomes across eight developmental stages (April through November) revealed the presence of 16 volatile components, comprising 14 sesquiterpenes, one monoterpene, and one polyacetylene (**Supplementary Table S2**). The cumulative relative content of the three primary active constituents—hinesol, β-eudesmol, and atractylodin—ranged from 76.19% to 86.11% (**Supplementary Figure S1A**). Furthermore, elemol and α-eudesmol were identified as relatively abundant sesquiterpenes, with relative contents ranging from 2.93% to 5.99% and 1.10% to 2.40%, respectively.

Heatmap analysis of these five major components indicated that their concentrations were lowest in June, increased to a peak in July, and subsequently declined from August onward. Notably, three of these components (hinesol, α-eudesmol, and atractylodin) exhibited a secondary increase in September before reaching their lowest levels in November (**Supplementary Figure S1B**). These findings underscore significant variations in the accumulation of major volatile compounds in *A. lancea* across different developmental stages, with pronounced fluctuations observed in June, July, September, and November.

### Developmental stages drive distinct metabolomic profiles and terpenoid accumulation dynamics

A metabolomic analysis was conducted on samples from four distinct developmental stages (June, July, September, and November) that demonstrated significant differences. Utilizing HS-SPME-GC-MS, a comprehensive total of 677 compounds were identified in *A. lancea* (Da Bie Shan type) across these stages, comprising 178 terpenoids, 121 esters, 87 heterocyclic compounds, 52 ketones, and 47 alcohols (**Supplementary Figure S2A**).

**Figure 1:**
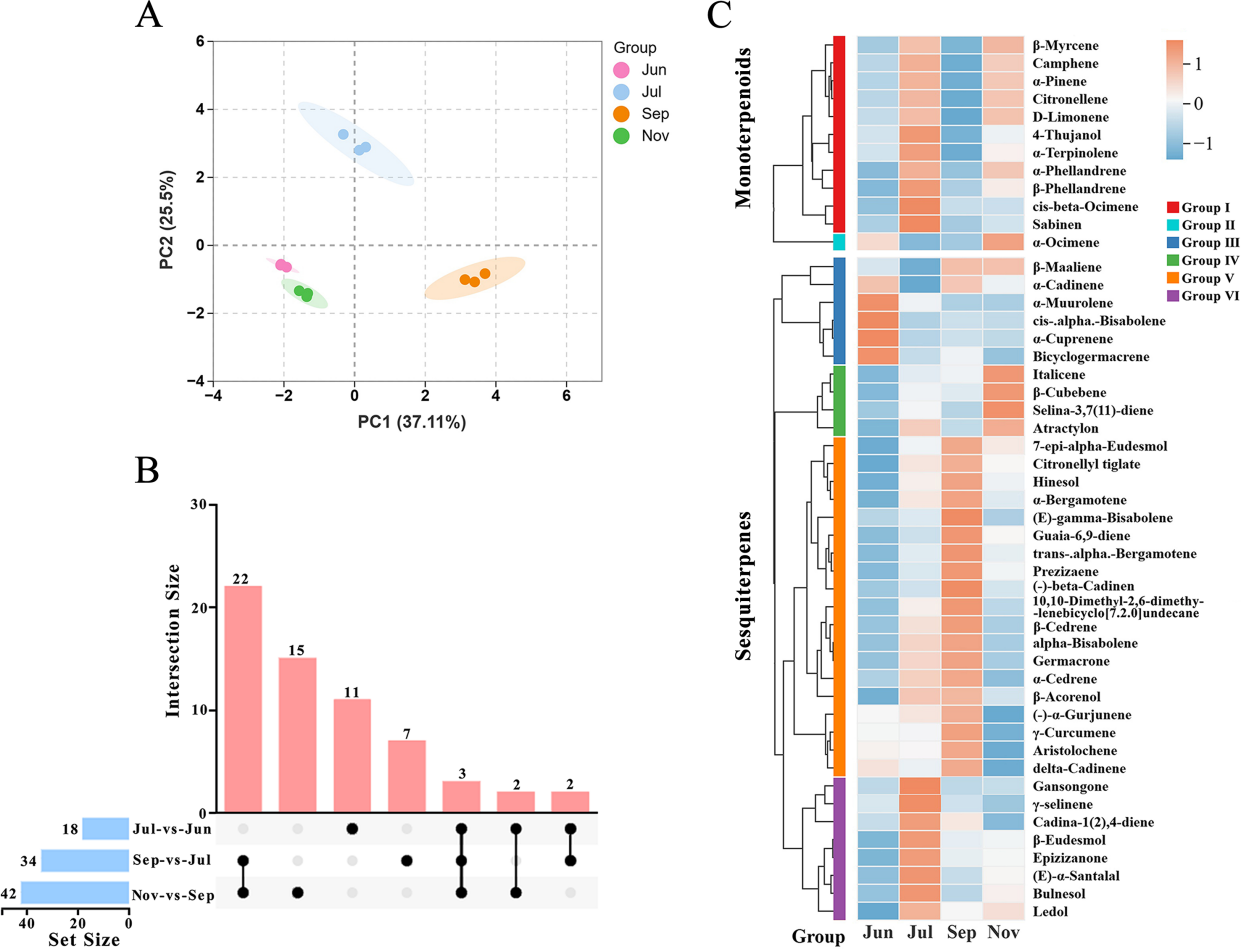
Analysis of terpenoid metabolites in *A. lancea* across four developmental stages. **(A)** Principal component analysis (PCA) of metabolites. **(B)** Heatmap analysis of the top 50 most abundant terpenoid compounds. **(C)** Differential terpenoid metabolites in different comparison groups.

To further elucidate the variations in metabolite accumulation patterns throughout these developmental stages, principal component analysis (PCA) was employed (**Figure 1A**). The PCA results revealed that principal components 1 and 2 accounted for 37.11% and 25.50% of the total variance, respectively, and effectively distinguished samples from the different developmental stages. The three biological replicates for each stage exhibited close clustering, indicating high data reliability and substantial metabolic differences between the stages.

A total of 50 terpenoid compounds with the highest abundance were selected to compare variations across developmental stages, comprising 38 sesquiterpenes and 12 monoterpenes (**Figure 1B**). Cluster analysis of these terpenoid compounds revealed distinct segregation patterns: the 12 monoterpenes were divided into two subgroups, while the 38 sesquiterpenes formed four subgroups. Within the monoterpenes, 11 compounds in Group I exhibited highly consistent accumulation dynamics, with peak levels observed in July and November and the lowest levels in June and September. Conversely, the single compound in Group II displayed a distinctly different accumulation pattern. For the sesquiterpenes, Groups III and IV reached maximum abundance in June and November, respectively, whereas Groups V and VI peaked in July and September. Notably, heatmap analysis of hinesol and β-eudesmol, both recognized as key bioactive constituents of *A. lancea*, indicated that hinesol concentration peaked in September, whereas β-eudesmol accumulation was most pronounced in July. These observations are corroborated by the GC-MS results. In summary, metabolite accumulation in *A. lancea* exhibited significant variation across different developmental stages.

A differential metabolite analysis was performed using adjacent developmental stages as comparative groups, with differential metabolites being identified for each group based on a variable importance in projection (VIP) score greater than 1. The analysis revealed that the July versus June comparison group identified 84 differential metabolites, comprising 54 upregulated and 30 downregulated metabolites. In the September versus July comparison group, 148 differential metabolites were identified, with 28 upregulated and 120 downregulated. The November versus September comparison group identified 184 differential metabolites, of which 143 were upregulated and 41 were downregulated (**Supplementary Figure 2B**). Further metabolomic analysis elucidated distinct stage-specific patterns in terpenoid biosynthesis. Specifically, monoterpenoids, exemplified by D-limonene, exhibited significant accumulation in July, whereas sesquiterpenoids, represented by β-elemene and germacrene D, became predominant in September. This observation collectively illustrates a dynamic shift in the terpenoid backbone flux from monoterpenes to sesquiterpenes (**Figure 1B**). KEGG enrichment analysis demonstrated that these differentially accumulated metabolites were predominantly enriched in two pathways: the biosynthesis of secondary metabolites (ko01110) and metabolic pathways (ko01100) (**Supplementary Figure S3**). Furthermore, an analysis of the terpenoid components among the differential metabolites in the three comparison groups was conducted. The July versus June group identified 18 terpenoid components, the September versus July group identified 34, and the November versus September group identified 42 terpenoid components (**Figure 1C**).

### Transcriptional landscape reveals stage-specific regulation of terpenoid biosynthesis pathways

To achieve a comprehensive understanding of the regulatory mechanisms governing terpenoid biosynthesis in *A. lancea* across various developmental stages, transcriptome identification and quantification were conducted utilizing RNA samples from each stage, with three biological replicates per stage. A total of 12 libraries were constructed, corresponding to the months of June, July, September, and November, each represented in triplicate, resulting in approximately 6.3–6.4 Gb of clean data per sample. The four developmental stages yielded 42.59, 42.44, 42.30, and 42.56 million clean reads, respectively, with Q30 values exceeding 93.82%. The average alignment rate to the reference genome was 93.22%, with 65.83% of the reads successfully mapped to the gene set. Quality assessment of RNA extracted from the rhizomes of *A. lancea* at the four stages indicated that all quality indicators, including RNA concentration, RIN value, and 28S/18S ratio, conformed to the required standards (**Supplementary Table S3**). The FPKM distribution boxplot (**Supplementary Figure 4A**) and expression density plots (**Supplementary Figure S4B**) depict the dispersion of gene expression levels and the range of expression abundance, respectively. Overall, the findings demonstrate a high sequencing quality that is well-suited for subsequent analyses.

**Figure 2:**
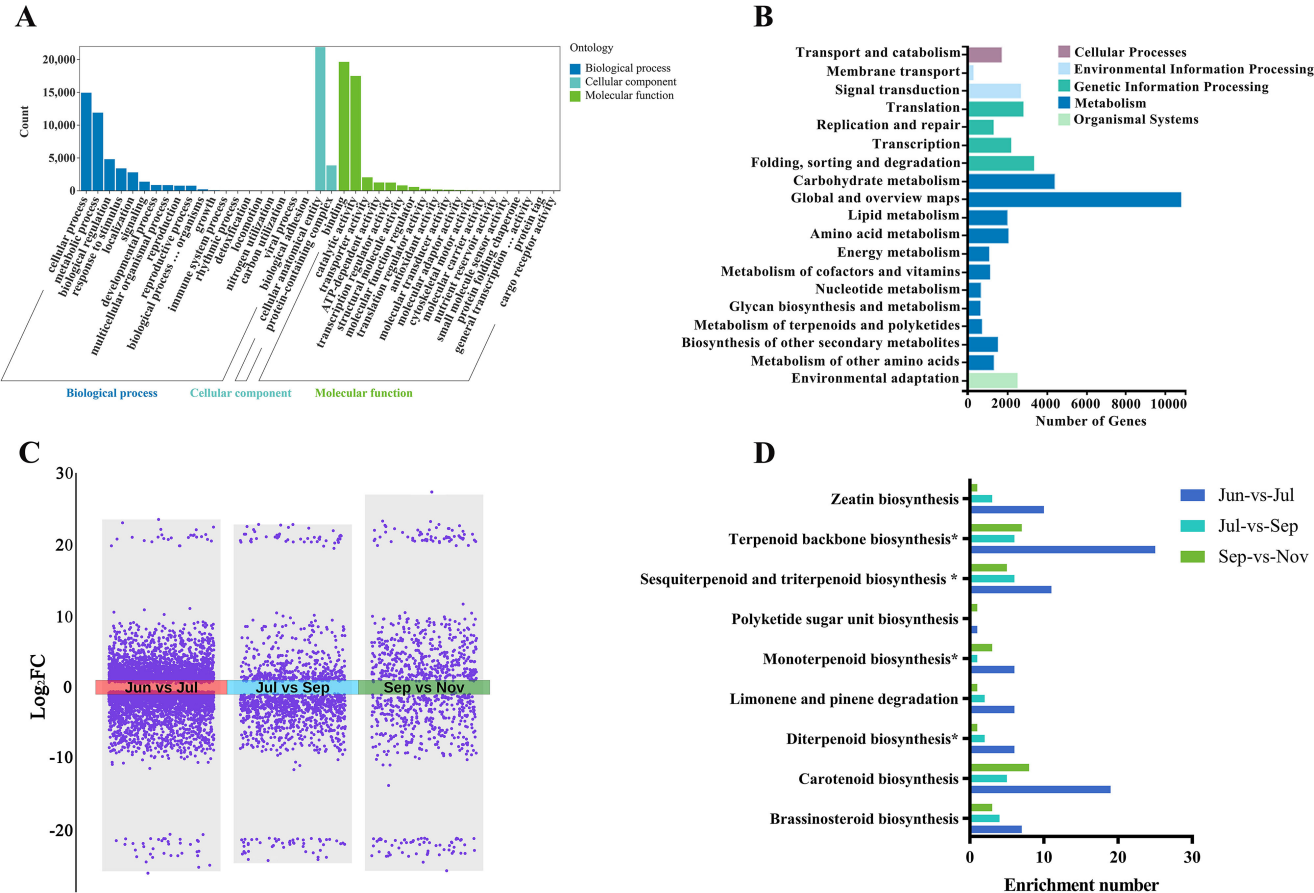
Functional annotation and DEG analysis of transcriptome genes across four developmental stages. **(A)** Gene Ontology (GO) functional annotation. **(B)** Kyoto Encyclopedia of Genes and Genomes (KEGG) functional annotation. **(C)** Volcano plots of DEGs in three comparison groups: Jun-vs-Jul, Jul-vs-Sep, and Sep-vs-Nov. **(D)** KEGG enrichment of DEGs involved in terpenoid and polyketide metabolism across the three comparison groups.

In the Gene Ontology (GO) functional annotation, a total of 34,551 genes were annotated and categorized into 41 terms spanning three principal categories: Biological Process, Cellular Component, and Molecular Function. The Biological Process category encompassed 20 terms, with “cellular process” being the most populous, comprising 14,944 genes. The Cellular Component category was limited to two terms: “cellular anatomical entity” and “protein-containing complex.” Within the Molecular Function category, which included 19 terms, “binding” emerged as the most prevalent function, represented by 19,642 genes, followed by “catalytic activity” with 17,499 genes (**Figure 2A**).

Regarding the KEGG functional annotation, 20,578 genes were classified into five primary categories and 19 subcategories based on KEGG pathways. The five major categories were Cellular Processes, Environmental Information Processing, Genetic Information Processing, Metabolism, and Organismal Systems. Among the subcategories, “Global and overview maps,” “Carbohydrate metabolism,” and “Folding, sorting, and degradation” contained the highest numbers of annotated genes. The subcategory “Metabolism of terpenoids and polyketides” was annotated with 722 genes (**Figure 2B**).

### Stage-specific gene expression activates terpenoid biosynthesis

To examine the dynamic patterns of gene expression across four developmental stages (June vs. July, July vs. September, September vs. November), stage-specific differentially expressed genes (DEGs) were identified. The comparison between June and July revealed 4,563 DEGs, with 2,062 genes up-regulated and 2,501 down-regulated. In contrast, the comparisons between July and September, and September and November, identified 1,318 DEGs (447 up-regulated, 871 down-regulated) and 893 DEGs (479 up-regulated, 414 down-regulated), respectively (**Figure 2C**). Venn diagram analysis identified 34 DEGs common to all three comparisons, while 3,992, 800, and 606 unique DEGs were identified in the June vs. July, July vs. September, and September vs. November comparisons, respectively (**Supplementary Figure S5**).

KEGG enrichment analysis indicated significant enrichment in the “Plant-pathogen interaction,” “Plant hormone signal transduction,” and “MAPK signaling pathway” across all comparisons (**Supplementary Figures S6A-C**). In the “Metabolism of terpenoids and polyketides” category, differentially expressed genes (DEGs) were significantly enriched across nine pathways, including “Terpenoid backbone biosynthesis,” “Monoterpenoid biosynthesis,” and “Sesquiterpenoid and triterpenoid biosynthesis.” The comparative analysis between June and July revealed a substantially higher number of DEGs in both the “Terpenoid backbone biosynthesis” and “Sesquiterpenoid and triterpenoid biosynthesis” pathways compared to other temporal comparisons, suggesting the activation of key terpenoid biosynthetic genes commencing in July. This observation is consistent with the detected accumulation patterns of monoterpenoid metabolites, as illustrated in **Figure 2D**.

### Integrated metabolomic and transcriptomic analysis uncovers key genes governing sesquiterpenoid biosynthesis

**Figure 3:**
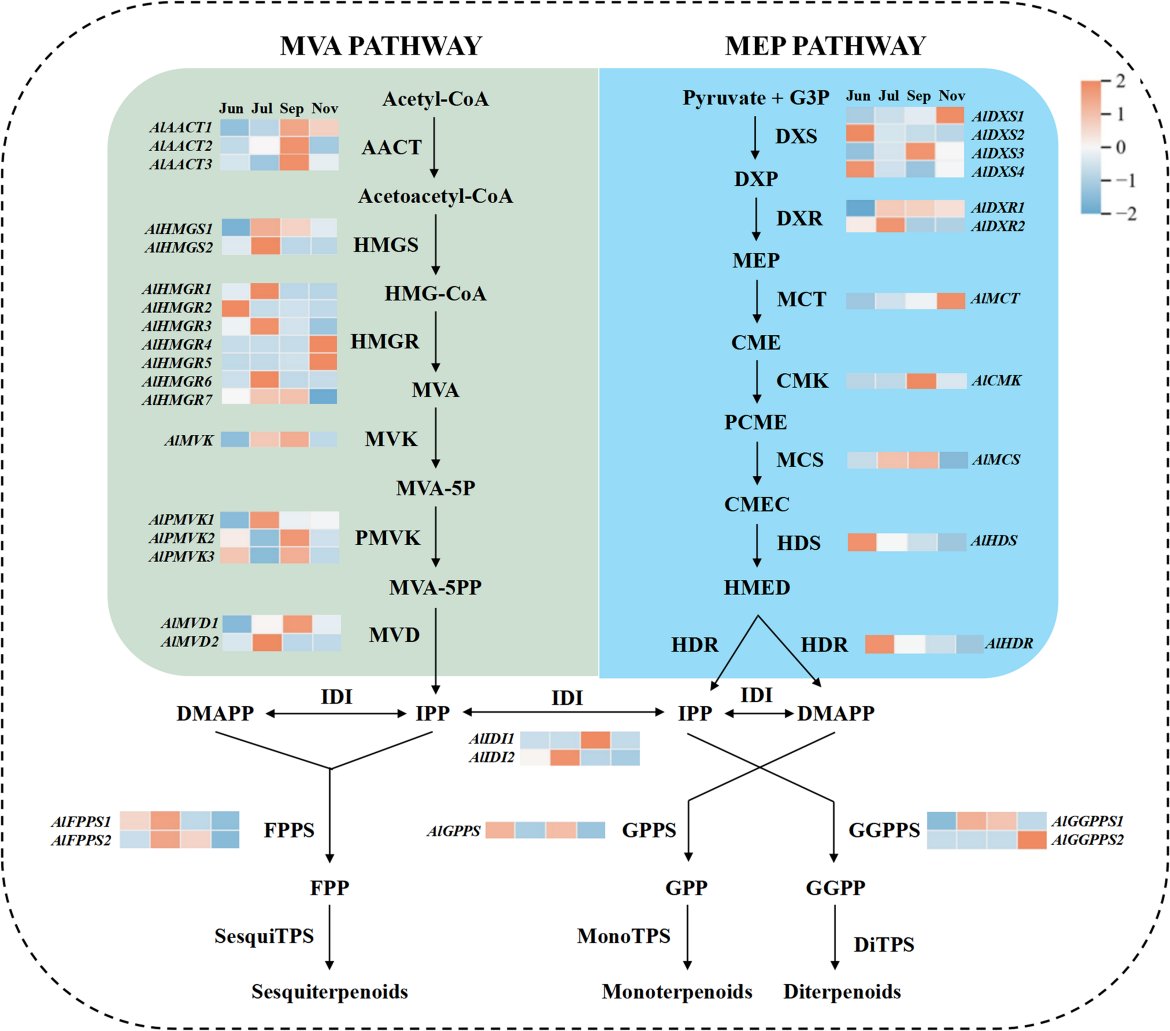
The biosynthetic pathways of terpenoids and the heat map of 36 genes related to the synthetic pathways.

An analysis was conducted on the pathways associated with terpenoid biosynthesis, as illustrated in **Figure 3**. In plants, the mevalonate (MVA) and methylerythritol phosphate (MEP) pathways represent the two principal metabolic routes for terpenoid biosynthesis. Within the MVA pathway, 18 genes encoding six key enzymes—AACT, HMGS, HMGR, MVK, PMVK, and MVD—were identified. Notably, seven genes (HMGS1/2, HMGR1/3/6, PMVK1, and MVD2) exhibited significantly elevated expression levels in July, while six genes (AACT1/2/3, PMVK2/3, and MVD1) were highly expressed in September. This suggests an enhanced activity of the MVA pathway during these months, which may be linked to the accumulation of sesquiterpenoids. In the MEP pathway, 11 genes encoding seven key enzymes (DXS, DXR, MCT, CMK, MDS, HDS, and HDR) were identified. Three genes (DXS2/4 and HDS) demonstrated high expression in June, whereas another three (DXR1/2 and MDS) were highly expressed in July, potentially contributing to monoterpenoid accumulation during this period.

The enzyme isopentenyl diphosphate isomerase (IDI) facilitates the interconversion of isopentenyl pyrophosphate (IPP) and dimethylallyl pyrophosphate (DMAPP), while geranyl pyrophosphate synthase (GPPS), farnesyl pyrophosphate synthase (FPPS), and geranylgeranyl pyrophosphate synthase (GGPPS) serve as branch-point enzymes essential for the biosynthesis of monoterpenes, sesquiterpenes, and diterpenes, respectively. In this study, we identified two IDI genes, two FPPS genes, one GPPS gene, and two GGPPS genes. Notably, *IDI2*, *FPPS1/2*, and GGPPS1 exhibited high expression levels in July, suggesting their significant roles in monoterpene and sesquiterpene synthesis during this period. The expression levels of these 36 genes associated with the terpenoid biosynthesis pathway are detailed in **Supplementary Table S4**. Furthermore, 55 terpene synthase (TPS) genes, including those encoding α-farnesene synthase, nerolidol synthase, and (-)-germacrene D synthase, were identified within terpenoid biosynthesis-related pathways (**Supplementary Table S5**).

A correlation analysis was performed to investigate the relationships between the 50 most abundant metabolites across four developmental stages and 36 genes implicated in terpenoid biosynthesis pathways. The analysis identified significant regulatory interactions, with 42 terpenoid compounds exhibiting notable correlations with 31 genes, thereby uncovering distinct patterns of metabolic regulation (P < 0.05) (**Supplementary Figure S7**). Notably, the expression level of *AlPMVK1* demonstrated a significant positive correlation with β-eudesmol content. Similarly, *AlMVD1* expression was significantly positively correlated with hinesol content, whereas *AlDXS4* expression showed a negative correlation with hinesol content. Additionally, *AlGPPS* expression was negatively correlated with atractylon content. These robust associations between upstream genes and terpenoid metabolites indicate that these genes are pivotal in the biosynthesis of key sesquiterpenoids in *A. lancea*. This underscores the necessity for further functional studies on downstream TPS genes and their regulatory effects on metabolites.

Quantitative reverse transcription PCR (qRT-PCR) validation was conducted on six selected genes from the terpenoid biosynthesis pathways. The expression patterns of these genes across four developmental stages were largely consistent with their fragments per kilobase of transcript per million mapped reads (FPKM) values (**Supplementary Figure S8**), thereby confirming the reliability of the transcriptome sequencing data.

### Terpene synthase gene discovery in *A. lancea*: *AlTPS21* and *AlTPS42* as multiproduct sesquiterpene synthases

**Figure 4:**
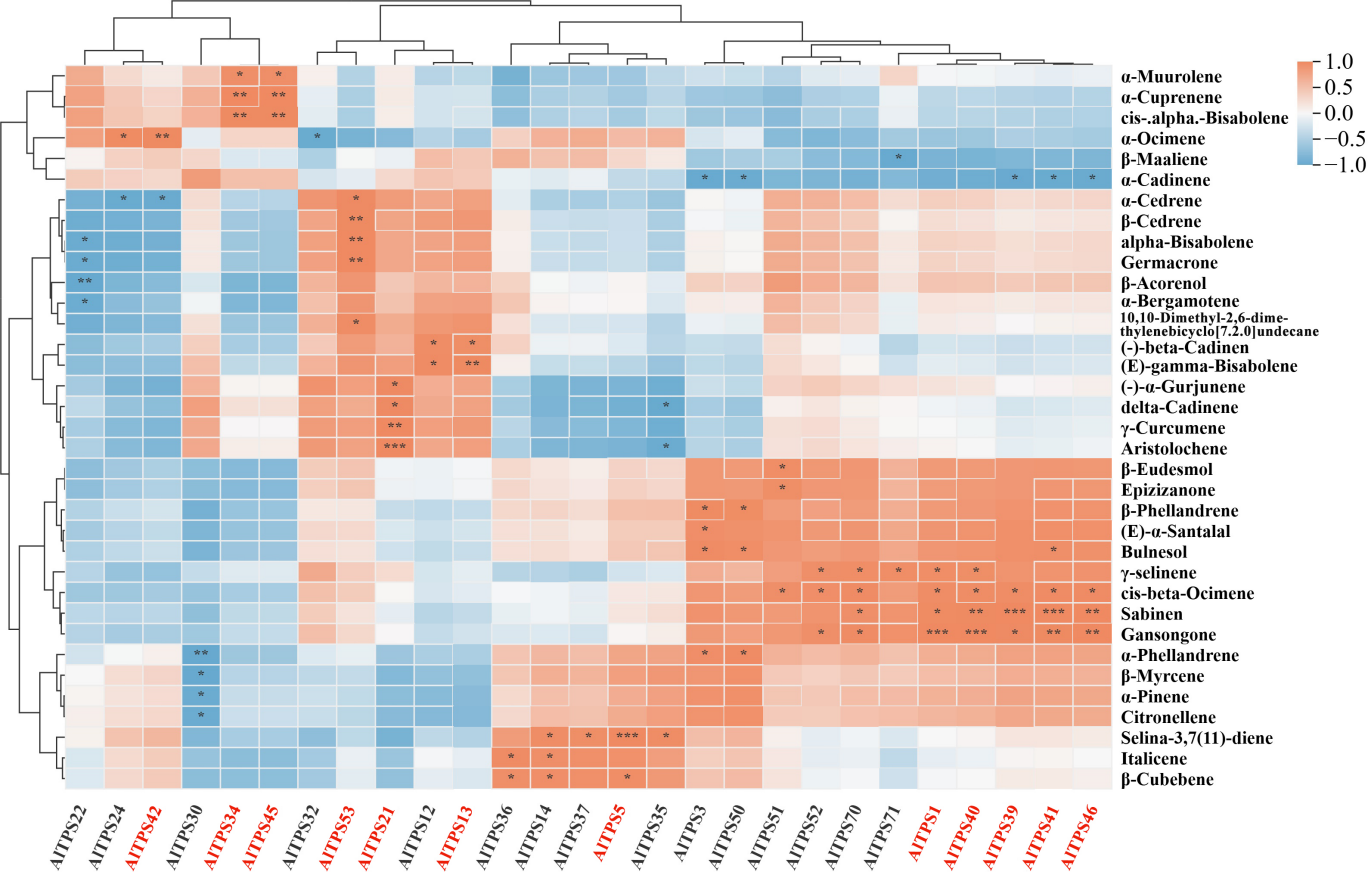
Correlation analysis between the top 50 most abundant terpenoid compounds and 33 AlTPS genes. Note: Orange indicates a positive correlation, blue indicates a negative correlation; significance levels are denoted by asterisks: * for 0.01 < p < 0.05, ** for 0.001 < p < 0.01, and *** for p ≤ 0.001.

A comprehensive analysis of the transcriptome data led to the identification of 55 AlTPSs, which were categorized into 33 TPS-a, 11 TPS-b, 2 TPS-e/f, and 9 TPS-g family members (**Supplementary Figure S9**; **Supplementary Tables S6-7**). Given that members of the TPS-a subfamily are predominantly involved in sesquiterpene synthesis, the 33 TPS-a genes were prioritized for an in-depth investigation into the mechanisms underlying sesquiterpenoid biosynthesis in *A. lancea*. A correlation analysis was conducted between these TPS-a genes and the top 50 most abundant terpenoid compounds. The findings indicated that 27 genes exhibited significant correlations with 35 terpenoid compounds (**Figure 4**). Notably, *AlTPS1* and *AlTPS40* demonstrated a highly significant positive correlation with Gansongone (P < 0.001). Furthermore, *AlTPS5* was found to be highly significantly positively correlated with Selina-3,7(11)-diene (P < 0.001), while *AlTPS13* showed a significantly positive correlation with (E)-gamma-Bisabolene (P < 0.01). *AlTPS21* exhibited a highly significant positive correlation with Aristolochene (P < 0.001), a significant positive correlation with γ-Curcumene, and positive correlations with δ-Cadinene and (-)-α-Gurjunene. The study identified significant positive correlations between specific terpene synthase genes and terpenoid components. *AlTPS34* and *AlTPS45* were strongly correlated with α-Cuprenene and cis-α-Bisabolene (P < 0.01), while *AlTPS39* and *AlTPS41* exhibited highly significant correlations with Sabinene (P < 0.001). *AlTPS42* showed a significant positive correlation with α-Ocimene (P < 0.01). Additionally, *AlTPS46* demonstrated significant correlations with both Sabinene and Gansongone. *AlTPS53* was significantly correlated with β-Cedrene, α-Bisabolene, and Germacrone (P < 0.01). In total, 12 terpene synthase genes were significantly associated with various terpenoid components.

Further analysis of the conserved protein motifs in 12 terpene synthase genes was conducted using the MEME online tool. The analysis identified that *AlTPS45* and *AlTPS53* lack the “RRX8W” motif (motif 11), which is crucial for cyclization. Additionally, the “NSE/DTE” motif (motif 7), which plays a role in metal-dependent ionization and substrate binding, was absent in *AlTPS13* and *AlTPS41*. Following a screening process, eight protein sequences containing complete conserved motifs were identified: *AlTPS1*, *AlTPS5*, *AlTPS21*, *AlTPS34*, *AlTPS39*, *AlTPS40*, *AlTPS42*, and *AlTPS46*, as detailed in **Supplementary Figure S10**.

**Figure 5:**
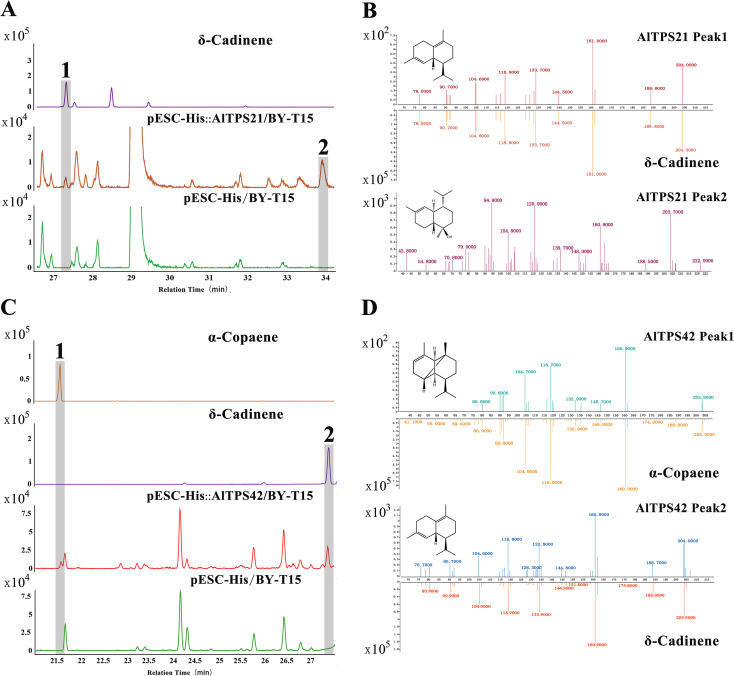
GC–MS analysis of catalytic products of AlTPS21 and AlTPS42. **(A)** Reaction products of AlTPS21 and the pESC-HIS empty vector. GC detection of the standard compound δ-cadinene. **(B)** MS analysis of the catalytic product of pESC-HIS-AlTPS21 and the δ-cadinene standard at 27.392 min; MS identification of AlTPS21 Peak2 confirmed it as α-cadinol. **(C)** Reaction products of AlTPS42 and the pESC-HIS empty vector. GC detection of standard compounds δ-cadinene and α-copaene. **(D)** MS analysis of the catalytic product of pESC-HIS-AlTPS42 and the α-copaene standard at 21.562 min, and MS analysis of the catalytic product and δ-cadinene standard at 27.406 min.

Following the successful identification of eight candidate genes from the TPS-a subfamily, we cloned four of these genes (*AlTPS1*, *AlTPS21*, *AlTPS40*, *AlTPS42*) into the pESC-His vector for functional characterization. The remaining four candidate genes (*AlTPS5*, *AlTPS34*, *AlTPS39*, *AlTPS46*) were excluded from functional analysis due to unsuccessful cloning attempts, which precluded the acquisition of correct recombinant plasmids. Volatile metabolites from the yeast cultures were analyzed using headspace solid-phase microextraction gas chromatography-mass spectrometry (HS-SPME-GC-MS), with the pESC-His empty vector strain serving as a negative control. As illustrated in **Figure 5**, the expression of *AlTPS21* resulted in the production of δ-cadinene (peak 1) and α-cadinol (peak 2). In the case of *AlTPS42*, two sesquiterpenoid products were detected in the yeast system: δ-cadinene (peak 2) and α-copaene (peak 1). No products were identified for *AlTPS1* and *AlTPS40* under the same experimental conditions, as shown in **Supplementary Figure S11**. We propose that a plausible explanation for these findings is that the native substrates for these enzymes may not be the universal precursor farnesyl diphosphate (FPP) naturally produced by the BY-T15 yeast strain. Moreover, their catalytic activity might necessitate plant-specific cofactors that are absent in this heterologous expression system. These findings provide significant avenues for future research, indicating that functional validation should be conducted through in planta experiments or by utilizing optimized expression platforms.

The tertiary structures of AlTPS21 and AlTPS42 were predicted using SWISS-MODEL, employing the template A0A5P8H4P8.1.A. The sequence similarity scores were 77.21% for AlTPS21 and 75.37% for AlTPS42, with both models achieving a GMQE value of 0.93. Three-dimensional protein structures were constructed and visualized using PyMOL in cartoon representation. Conserved motifs were highlighted in colored stick models: the RRX8W motif in orange, DDXXD in green, NSE/DTE in yellow, and RXR in red, as depicted in **Supplementary Figure S12**.

### Subcellular localization of AlTPS gene

**Figure 6:**
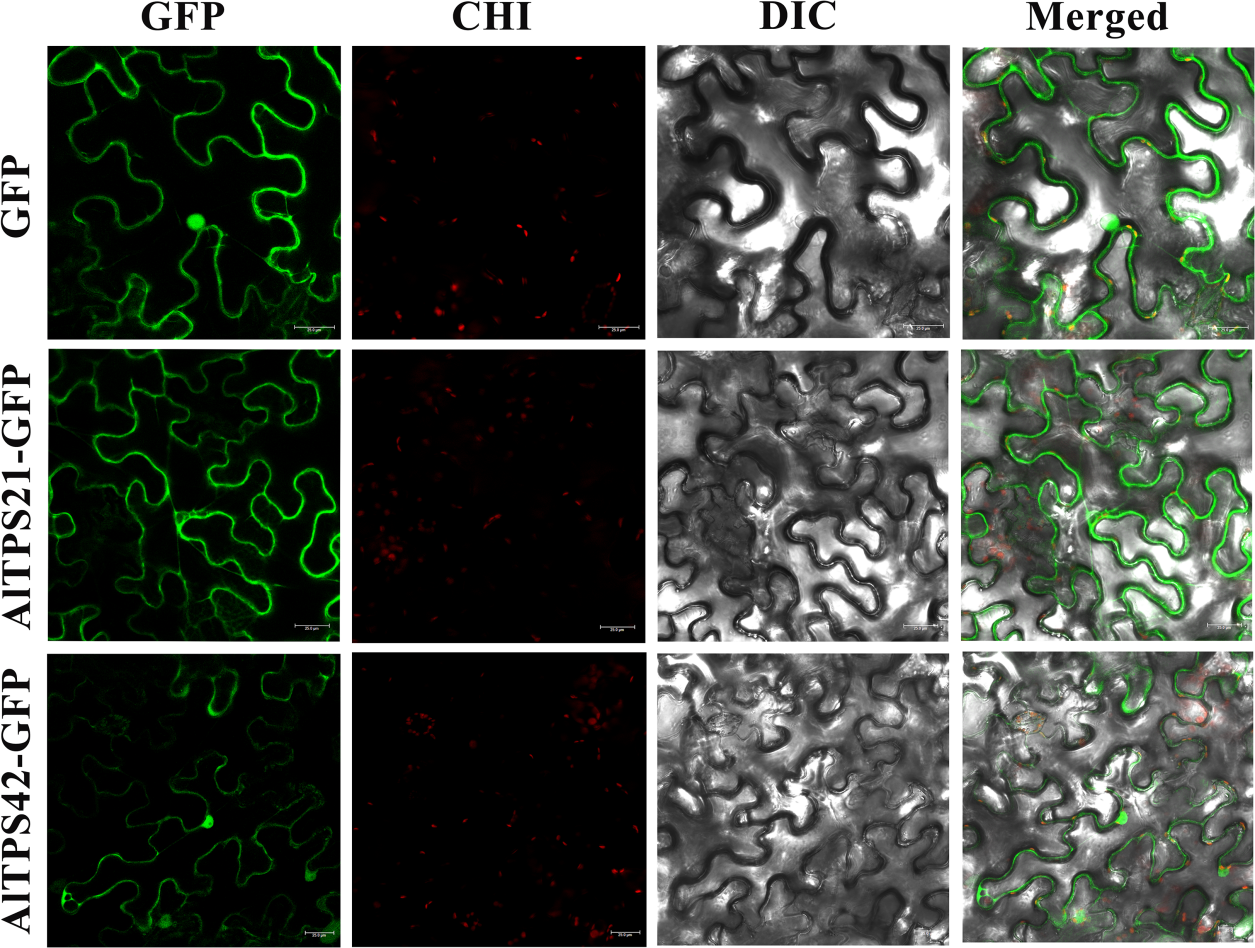
Subcellular localization of AlTPS21 and AlTPS42 proteins. GFP served as a negative control. Green fluorescence indicates the localization of the fusion proteins. Chlorophyll autofluorescence (CHI) shows the chloroplast channel. Merged images represent the overlay of GFP and chlorophyll signals. Scale bar = 25 μm.

Subcellular localization analysis was conducted through the construction of GFP-tagged expression vectors. The GFP fluorescence of AlTPS proteins, along with empty vector controls, was examined using confocal laser scanning microscopy. Microscopic analysis revealed that both AlTPS21 and AlTPS42 were localized to the nucleus and cell membrane (**Figure 6**). To further elucidate their subcellular localization, co-localization experiments were performed utilizing a chloroplast marker. Green fluorescence indicated the expression and distribution of the GFP-tagged proteins, while red fluorescence denoted the distribution of chloroplasts labeled by the marker. Regions of overlap between green and red fluorescence appeared yellow. The merged results demonstrated that the AlTPS21 protein was localized to the cell membrane, whereas AlTPS42 was localized to both the nucleus and the cell membrane.

## Discussion

Recent research on *A. lancea* has predominantly concentrated on its volatile oils and pharmacological properties (Peng et al., 2022; Qu et al., 2022), whereas comprehensive studies on the dynamic accumulation patterns of metabolites across various developmental stages are still scarce. Metabolomic analysis not only aids in identifying key signaling metabolites involved in regulatory processes within plants but also provides a crucial foundation for integrating multi-omics strategies to identify key genes in sesquiterpenoid biosynthesis pathways (Okada et al., 2010; Scossa et al., 2018). This study conducted an analysis of the metabolic profiles of *A. lancea* across various developmental stages, revealing distinct temporal patterns in terpenoid accumulation. Specifically, monoterpenoids exhibited a rapid increase in July, while sesquiterpenoids, which were initially low in June, began to accumulate in July, peaked in September, and subsequently declined by November. These findings illustrate clear dynamics dependent on developmental stages. Such stage-specific accumulation patterns are consistent with observations in other medicinal plants. For instance, Wang et al. (2024) reported that in the essential oil of *Citrus aurantium* peel, oxygenated monoterpenes, such as citronellol, peaked during mid-growth stages and subsequently decreased with maturation, whereas sesquiterpenes, like elemene, gradually increased during the ripening process. Similarly, research on *Magnolia biondii* flower buds (Hu et al., 2018) and *Satureja macrantha* essential oil across various phenological stages (Aghbash et al., 2020) has consistently demonstrated a significant influence of developmental stage on the composition and content of volatile oils. The findings of this study not only elucidate the dynamic accumulation patterns of terpenoids in the Dabieshan variety of *A. lancea* but also provide a scientific basis for determining optimal harvesting times. This study offers insights into the quality assessment and sustainable use of *A. lancea* medicinal materials.

Terpenoids, which are essential secondary metabolites in plants, are primarily synthesized through the cytoplasmic mevalonate (MVA) pathway and the plastidial 2-C-methyl-D-erythritol 4-phosphate (MEP) pathway (Ajikumar et al., 2010; Miziorko, 2011). A transcriptomic analysis of Dabieshan-type *A. lancea* across four developmental stages revealed a significant upregulation of MVA pathway genes, specifically HMGR and PMVK, during the months of July and September, coinciding with peaks in sesquiterpenoid accumulation. This observation supports the predominant role of the MVA pathway, as also documented by Zhao et al. (2013). Furthermore, the expression of HMGR exhibited a positive correlation with sesquiterpenoid levels, corroborating findings in *Panax ginseng* (Kim et al., 2014) and *Artemisia annua* (Aquil et al., 2009). In contrast, MEP pathway genes, including DXS and DXR, demonstrated heightened expression in June, facilitating the supply of monoterpene precursors. In this study, we identified two paralogs of DXR *(DXR1* and *DXR2*) that exhibited developmental stage-specific expression patterns, thereby extending the established role of DXR in the regulation of monoterpenes, as previously reported in mint (Mahmoud and Croteau, 2001) and lily (Zhang et al., 2018). The simultaneous increase in IDI2 expression alongside terpenoid production in July further substantiates the hypothesis of cross-pathway regulation (Bergman et al., 2019).

The TPS family is pivotal in terpenoid biosynthesis and is categorized into eight subfamilies (TPS-a to TPS-h) (Jia et al., 2022). Integrated transcriptomic and metabolomic analyses enable the identification of key genes by correlating metabolite accumulation with gene expression within relevant pathways (Liu et al., 2024a; Lin et al., 2020). Employing this methodology, we identified eight candidate genes, successfully cloned four, and conducted functional characterization of *AlTPS21* and *AlTPS42*. Notably, the expression peaks of *AlTPS21* and *AlTPS42* align with the peak of sesquiterpenoid accumulation in September, identifying them as crucial agents in translating developmental and environmental signals into specific metabolic phenotypes. Prior research on Asteraceae species, including *Artemisia annua* and *Xanthium strumarium*, has effectively characterized TPS enzymes through heterologous expression systems in *E. coli* and *S. cerevisiae* (Li et al., 2013, 2016). In the present study, expression in *S. cerevisiae* revealed that AlTPS21 synthesizes δ-cadinene and α-cadinol, whereas AlTPS42 produces δ-cadinene and α-copaene. This functional diversification, coupled with their stage-specific expression, directly influences the dynamic sesquiterpenoid flux and chemodiversity observed during rhizome development. Intriguingly, experimental results demonstrated that both AlTPS21 and AlTPS42 are localized in both the nucleus and the cell membrane. This unique distribution pattern suggests that these enzymes may have dual functional roles: potentially modulating gene activity within the nucleus while also participating in intercellular signaling at the membrane level. The coordinated activities at these two cellular sites appear to collectively impact the biosynthesis of sesquiterpenoid compounds. Although both enzymes generate δ-cadinene, their distinct product spectra highlight the catalytic promiscuity of TPSs and imply that functional variation may result from subtle differences in active site residues (Christianson, 2017). Our findings reveal significant parallels with the study on maize *ZmTPS8* by Saldivar et al. (2023), as both investigations underscore the catalytic versatility of plant terpene synthases. Specifically, ZmTPS8 has been demonstrated to produce δ-cadinene and α-copaene. In this study, we establish that AlTPS42 similarly catalyzes the formation of these sesquiterpenes, while AlTPS21 predominantly generates δ-cadinene and its oxygenated derivative, α-cadinol. This functional conservation across species provides valuable insights into the evolutionary mechanisms underpinning terpenoid-based defense metabolism in plants. Previous research has documented the pharmacological properties of these sesquiterpenes, including anticancer and insect-repellent activities (Magnani et al., 2025; Ferraz et al., 2013). The identification of these functionally characterized and ecologically significant genes lays the foundation for future endeavors aimed at enhancing the production of bioactive terpenoids through metabolic engineering, thereby advancing the development of plant-derived pharmaceuticals.

This study employed an integrated multi-omics approach to elucidate the temporal regulation of terpenoid biosynthesis in *A. lancea* rhizomes. Utilizing this methodology, we identified key structural and regulatory genes and successfully characterized the functional properties of two sesquiterpene synthases, AlTPS21 and AlTPS42. Our findings indicate that intrinsic developmental programming predominantly governs terpenoid accumulation patterns, as demonstrated by the stage-specific expression of biosynthetic pathway genes and terpenoid synthases. Nonetheless, the interaction between developmental programs and environmental factors warrants further exploration. For instance, the unique metabolic profile observed in September may result from both developmental timing and plant adaptations to early autumn changes in photoperiod and temperature (Zhang et al., 2021). Such environmental cues may modulate metabolic production through direct regulation of key gene expression or via phytohormone-mediated pathways, with jasmonic acid being a well-documented inducer of terpenoid biosynthesis (Zhang et al., 2025; He and Zhao, 2015). This integrated methodology, encompassing controlled environment experiments, multi-regional field monitoring, and concurrent phytohormone and genomic profiling, facilitates a systematic analysis of individual environmental factors and their interactions with gene networks. This comprehensive strategy aims to elucidate the fundamental principles underlying the formation of medicinal components in *A. lancea*.

## Conclusion

This study provides a comprehensive elucidation of the developmental regulatory mechanisms governing terpenoid biosynthesis in *A. lancea*, employing an integrated multi-omics approach coupled with functional validation. Our findings disclose distinct stage-specific accumulation patterns of terpenoids, with monoterpenoids and sesquiterpenoids peaking in July and September, respectively. Additionally, we demonstrate the temporal coordination between the mevalonate (MVA) and methylerythritol phosphate (MEP) pathways, highlighting their complementary regulatory roles in terpenoid biosynthesis. Notably, we have identified two novel terpene synthase genes, *AlTPS21* and *AlTPS42*, which exhibit unique catalytic specificity for the production of multiple sesquiterpenoids, with expression patterns that align with the peaks of sesquiterpenoid accumulation. Their atypical subcellular localization further suggests potential non-canonical roles in plant secondary metabolism. These findings enhance our understanding of the regulation of terpenoid biosynthesis in medicinal plants from a spatiotemporal perspective. The outcomes of this research identify specific targets for the molecular breeding of *A. lancea* and establish a robust research framework that extends from gene discovery to functional validation, offering valuable insights for the investigation of secondary metabolism in other medicinal plants.

## Data Availability

The datasets presented in this study can be found in online repositories. The names of the repository/repositories and accession number(s) can be found in the article/**Supplementary Material**.
