# Supplementary material for: Transcriptome and metabolome analysis of Atractylodes lancea across different developmental stages
Source: Front Plant Sci. 2025 Nov 28;16:1720423. doi: 10.3389/fpls.2025.1720423 (PMC12716082; doi:10.3389/fpls.2025.1720423)
Supplement: Supplementary file 1 [file Table1.docx]

Supplementary Table S1 The primers used for real-time quantitative PCR

| **Gene** | **Primer sequence（5’-3’）** | |
| --- | --- | --- |
|  | **F** | **R** |
| *AACT* (*AtL_chr01G0915*) | ACGGGTTGTGGGATGTTTTC | TCCTCTTGGGCCAGGAACT |
| *HMGS* (*AtL_chr01G4427*) | GCTGATTGGGCCTGATGCT | GGTCGGGCTTGTAGAAGTCGTA |
| *MVK* (*AtL_chr03G0702*) | CGCCTTGCTTGCTTTATCAG | TGTGCTGACTGTATTGTCTATCCC |
| *MVD* (*AtL_chr04G4117*) | TGGATTTGTCAAGTGGGTCATG | TGAGGTGCTACTGGTTTCTTTCTG |
| *DXR* (*AtL_chr03G3179*) | CTGGCTCAAACATCGCACTT | GGGCAACCTCAATGACACCT |
| *GGPPS* (*AtL_chr06G1893*) | TTGCTGGTGACGCTTTATTCC | AGGAGCTGGGTTTCTGGGAC |
| *AlUBQ2* | GGTTGAGGGGAGGAATGC | AGACGAAGGACAAGGTGA |

Supplementary Table S2 Identification of volatile components in rhizomes of *Atractylodes lancea* across different developmental stages.

| No. | RT | Molecular Formula | Compound | Relative Content±SD（%） | | | | | | | |
| --- | --- | --- | --- | --- | --- | --- | --- | --- | --- | --- | --- |
|  |  |  |  | Apr | May | Jun | Jul | Aug | Sep | Oct | Nov |
| 1 | 12.406 | C_10_H_16_ | α-Phellandrene | 0.05±0.03 | 0.02±0.003 | 0.04±0.033 | 0.13±0.098 | 0.12±0.105 | 0.2±0.036 | 0.66±0.248 | 0.93±0.269 |
| 2 | 27.191 | C_15_H_24_ | delta-Elemene | 0.02±0.01 | 0.02±0.01 | - | 0.02±0.003 | 0.03±0.01 | 0.02±0.021 | 0.02±0.005 | 0.04±0.066 |
| 3 | 29.801 | C_15_H_24_ | Berkheyaradulene | 0.15±0.11 | 0.15±0.04 | 0.15±0.04 | 0.1±0.03 | 0.14±0.05 | 0.22±0.18 | 0.09±0.06 | 0.27±0.06 |
| 4 | 31.116 | C_15_H_24_ | β-Caryophyllene | 0.19±0.12 | 0.19±0.05 | 0.17±0.04 | 0.15±0.03 | 0.19±0.02 | 0.29±0.19 | 0.13±0.04 | 0.27±0.06 |
| 5 | 32.626 | C_15_H_24_ | α-Humulene | 0.05±0.03 | 0.05±0.01 | 0.04±0.01 | 0.04±0.01 | 0.05±0.01 | 0.08±0.05 | 0.03±0.02 | 0.08±0.01 |
| 6 | 33.286 | C_15_H_24_ | β-Himachalene | 0.14±0.11 | 0.07±0.04 | 0.17±0.06 | 0.25±0.18 | 0.2±0.09 | 0.23±0.23 | 0.19±0.22 | - |
| 7 | 33.414 | C_15_H_22_ | Curcumene | 0.02±0.02 | - | 0.03±0.05 | 0.02±0.02 | 0.03±0.01 | 0.02±0.01 | - | - |
| 8 | 33.961 | C_15_H_24_ | Zingiberene | 0.003±0.003 | 0.039±0.019 | 0.393±0.631 | 0.034±0.039 | 0.147±0.093 | 0.11±0.118 | - | 0.124±0.215 |
| 9 | 35.205 | C_15_H_24_ | β-Sesquiphellandrene | 0.06±0.02 | 0.06±0.01 | 0.1±0.1 | 0.06±0.02 | 0.14±0.06 | 0.15±0.09 | 0.07±0.01 | - |
| 10 | 36.274 | C_15_H_26_O | Elemol | 5.35±2.56 | 5.36±2.24 | 3.97±1.86 | 4.84±2.55 | 5.99±0.36 | 3.37±2.8 | 2.93±1.72 | 3.07±0.82 |
| 11 | 38.234 | C_15_H_26_O | Guaiol | 0.05±0.01 | 0.08±0.05 | 0.08±0.02 | 0.08±0.01 | 0.07±0.03 | 0.09±0.05 | 0.07±0.02 | 0.04±0.05 |
| 12 | 39.048 | C_15_H_26_O | Cubenol | 0.28±0.03 | 0.26±0.01 | 0.25±0.1 | 0.23±0.06 | 0.27±0.04 | 0.26±0.08 | 0.27±0.02 | 0.25±0.09 |
| 13 | 40.215 | C_15_H_26_O | Hinesol | 47.63±6.38 | 47.17±2.79 | 45.99±10.45 | 39.5±5.65 | 38.08±5.09 | 52.2±3.94 | 49.16±4.05 | 45.28±2.47 |
| 14 | 40.855 | C_15_H_26_O | β-Eudesmol | 35.17±5.89 | 35±1.26 | 36.49±12.55 | 41.26±4.38 | 43.18±3.42 | 27.9±1.51 | 33.77±5 | 30.28±2.99 |
| 15 | 41.264 | C_15_H_26_O | α-Eudesmol | 1.43±0.6 | 1.78±0.67 | 1.7±0.25 | 2.31±1.38 | 1.74±0.74 | 2.4±0.87 | 1.77±0.29 | 1.1±0.18 |
| 16 | 46.617 | C_13_H_10_O | Atractylodin | 2.02±1.06 | 1.67±1 | 1.93±2.29 | 3.05±0.88 | 1.19±1.15 | 3.03±1.35 | 3.18±2.34 | 0.63±0.26 |

Supplementary Table S3. Quality assessment of sequencing data from *A. lancea* rhizomes.

| **Sample** | **Total Raw Reads (M)** | **Total Clean Reads (M)** | **Total Clean Bases**  **(Gb)** | **Clean Reads Q20**  **(%)** | **Clean Reads Q30**  **(%)** | **Clean Reads Ratio**  **(%)** | **Concentration**  **(ng/μL)** | **RIN** | **28S/18S** |
| --- | --- | --- | --- | --- | --- | --- | --- | --- | --- |
| Jun_1 | 43.69 | 42.5 | 6.37 | 98.22 | 93.82 | 97.27 | 865 | 8.7 | 1.4 |
| Jun_2 | 43.69 | 42.64 | 6.4 | 98.37 | 94.36 | 97.59 | 220 | 8.6 | 1.5 |
| Jun_3 | 43.69 | 42.62 | 6.39 | 98.28 | 94.09 | 97.56 | 240 | 8.1 | 1.6 |
| Jul_1 | 43.69 | 42.42 | 6.36 | 98.23 | 93.9 | 97.09 | 360 | 9.3 | 1.6 |
| Jul_2 | 43.69 | 42.46 | 6.37 | 98.27 | 94.03 | 97.18 | 445 | 9 | 1.5 |
| Jul_3 | 43.69 | 42.45 | 6.37 | 98.24 | 93.93 | 97.15 | 540 | 8.9 | 1.5 |
| Sep_1 | 43.69 | 42.4 | 6.36 | 98.32 | 94.19 | 97.04 | 194 | 8.2 | 1.2 |
| Sep_2 | 43.69 | 41.98 | 6.3 | 98.33 | 94.23 | 96.08 | 330 | 8.8 | 1.4 |
| Sep_3 | 43.69 | 42.53 | 6.38 | 98.28 | 94.05 | 97.34 | 147 | 8.4 | 1.3 |
| Nov_1 | 43.69 | 42.66 | 6.4 | 98.28 | 94.06 | 97.65 | 140 | 9.2 | 1.6 |
| Nov_2 | 43.69 | 42.51 | 6.38 | 98.36 | 94.25 | 97.31 | 330 | 8.3 | 1.1 |
| Nov_3 | 43.69 | 42.51 | 6.38 | 98.34 | 94.26 | 97.29 | 134 | 8.8 | 1.7 |

Supplementary Table S4 FPKM of genes related to terpenoid backbone biosynthesis in *A. lancea* at four developmental stages

| **Gene** | **ID** | **FPKM±SD** | | | |
| --- | --- | --- | --- | --- | --- |
|  |  | **Jun** | **Jul** | **Sep** | **Nov** |
| AACT | *AtL_chr01G0915* | 21.84±9.77 | 28.64±12.8 | 57.55±39.12 | 47.82±12.8 |
|  | *AtL_chr01G3973* | 55.94±11.05 | 69.11±50.03 | 99.75±73.74 | 48.99±19.2 |
|  | *AtL_chr02G6090* | 68.69±6.75 | 58.03±3.94 | 102.02±46.23 | 71.96±13.21 |
| HMGS | *AtL_chr01G4427* | 8.46±1.78 | 39.64±23.23 | 32.83±21.52 | 22.84±8.2 |
|  | *AtL_chr12G1942* | 12.07±5.43 | 14.51±13.79 | 14.74±13.79 | 6.94±3.06 |
| HMGR | *AtL_chr04G2198* | 28.94±20.91 | 167.56±131.8 | 1.21±1.73 | 0.69±0.99 |
|  | *AtL_chr06G2955* | 21.01±6.49 | 96.85±59.26 | 3.56±5.55 | 1.69±2.48 |
|  | *AtL_chr08G3097* | 21.82±7.48 | 8.12±1.92 | 8.83±4.56 | 7.78±0.74 |
|  | *AtL_chr09G3467* | 2.53±1.37 | 6.46±3.6 | 1.8±1.79 | 0.33±0.05 |
|  | *AtL_chr10G0183* | 0±0 | 0±0 | 0±0 | 0.02±0.03 |
|  | *AtL_chr11G2442* | 0.05±0.05 | 0.46±0.42 | 2.32±3.95 | 30.25±49.21 |
|  | *AtL_chr11G2445* | 6.68±9.12 | 117.13±141.76 | 0.76±0.72 | 3.14±5.24 |
| MVK | *AtL_chr03G0702* | 4.42±1.17 | 10.63±4.23 | 12.04±6.66 | 6.15±1.45 |
| PMVK | *AtL_chr01G5922* | 4.95±0.42 | 20.45±3.88 | 11.35±3.42 | 11.88±3.78 |
|  | *AtL_chr08G1096* | 7.39±4.72 | 3.87±1.7 | 10.72±10.25 | 5.66±2.77 |
|  | *AtL_chr08G1146* | 11.97±3.37 | 3.55±1.48 | 13.38±9.52 | 6.12±2.65 |
| MVD | *AtL_chr04G4117* | 23.8±6.35 | 42.36±14 | 61.35±11.8 | 39.2±14.57 |
|  | *AtL_chr09G3650* | 11.97±10.76 | 76.28±125.28 | 1.94±1.05 | 3.24±0.52 |
| DXS | *AtL_chr01G7881* | 9.05±1.35 | 9.26±1.66 | 9.41±0.8 | 10.47±0.93 |
|  | *AtL_chr06G2729* | 43.56±27.32 | 17.69±6.92 | 15.41±7.22 | 13.72±2.52 |
|  | *AtL_chr07G2906* | 0.07±0.12 | 1.35±1.96 | 4.64±7.33 | 2.06±3.46 |
|  | *AtL_chr11G1557* | 9.42±8.01 | 4.56±2.47 | 3.11±2 | 5.55±2.15 |
| DXR | *AtL_chr03G3179* | 15.91±3.2 | 49.85±24.89 | 48.69±16.68 | 45.32±3.18 |
|  | *AtL_chr05G2329* | 6.94±5.13 | 10.78±3.18 | 3.82±0.78 | 3.98±3.06 |
| MCT | *AtL_chr07G2022* | 13.18±0.59 | 17.05±2.16 | 19.65±2.99 | 31.45±5.61 |
| CMK | *AtL_chr04G1122* | 16.09±0.82 | 16.68±4.13 | 35.94±17.99 | 19.14±4.1 |
| MDS | *AtL_chr08G0576* | 17.36±3.23 | 22.15±4.91 | 22.95±10.71 | 14.8±6.63 |
| HDS | *AtL_chr04G3543* | 131.83±36.49 | 84.92±19.61 | 69.98±28.97 | 54.76±2.05 |
| HDR | *AtL_chr07G1219* | 97.17±13.95 | 93.49±23.44 | 148.01±58 | 86.34±11.23 |
| IDI | *AtL_chr04G0784* | 72.57±25.78 | 72.25±7.87 | 121.19±87.38 | 71.06±21.04 |
|  | *AtL_chr08G1672* | 48.13±20.7 | 81.11±82.69 | 31.44±12.37 | 26.84±1.39 |
| FPPS | *AtL_chr01G0080* | 2.18±1.11 | 2.91±3.18 | 1.12±1 | 0.63±0.43 |
|  | *AtL_chr05G2895* | 31.98±20.42 | 59.06±78.87 | 48.12±40.76 | 20.61±9.59 |
| GPPS | *AtL_chr07G3217* | 11.11±6.15 | 4.29±1.14 | 10.57±4.21 | 3.58±0.82 |
| GGPPS | *AtL_chr06G1893* | 12.48±1.56 | 35.06±18.27 | 32.22±4.54 | 18.4±5.16 |
|  | *AtL_chr06G2741* | 0±0 | 0±0 | 0±0 | 0.27±0.47 |

Supplementary Table S5 Key genes involved in the terpenoid biosynthesis pathways in *A. lancea.*

| Enzyme Abbreviation | EC number | Gene number |
| --- | --- | --- |
| acetyl-CoA C-acetyltransferase (AACT) | 2.3.1.9 | 3 |
| hydroxymethylglutaryl-CoA synthase (HMGS) | 2.3.3.10 | 2 |
| hydroxymethylglutaryl-CoA reductase (HMGR) | 1.1.1.34 | 7 |
| mevalonate kinase (MVK) | 2.7.1.36 | 1 |
| phosphomevalonate kinase (PMVK) | 2.7.4.2 | 3 |
| diphosphomevalonate decarboxylase (MVD) | 4.1.1.33 | 2 |
| 1-deoxy-D-xylulose-5-phosphate synthase (DXS) | 2.2.1.7 | 4 |
| 1-deoxy-D-xylulose-5-phosphate reductoisomerase (DXR) | 1.1.1.267 | 2 |
| 2-C-methyl-D-erythritol 4-phosphate cytidylyltransferase (MCT) | 2.7.7.60 | 1 |
| 4-diphosphocytidyl-2-C-methyl-D-erythritol kinase (CMK) | 2.7.1.148 | 1 |
| 2-C-methyl-D-erythritol 2,4-cyclodiphosphate synthase (MDS) | 4.6.1.12 | 1 |
| (E)-4-hydroxy-3-methylbut-2-enyl-diphosphate synthase (HDS) | 1.17.7.1 | 1 |
| 4-hydroxy-3-methylbut-2-en-1-yl diphosphate reductase (HDR) | 1.17.7.4 | 1 |
| isopentenyl-diphosphate Delta-isomerase (IDI) | 5.3.3.2 | 2 |
| farnesyl diphosphate synthase (FPPS) | 2.5.1.10 | 2 |
| geranyl diphosphate synthase (GPPS) | 2.5.1.1 | 1 |
| geranylgeranyl diphosphate synthase (GGPPS) | 2.5.1.29 | 2 |
| Terpene Synthase (TPS) | 4.2.3.27 | 4 |
|  | 4.2.3.27, 4.2.3.48 | 5 |
|  | 4.2.3.46 | 1 |
|  | 4.2.3.48 | 4 |
|  | 4.2.3.13, 4.2.3.75 | 16 |
|  | 4.2.3.75 | 17 |
|  | 4.2.3.27,4.2.3.111 | 6 |
|  | 4.2.3.19 | 2 |

Supplementary Table S6 Correspondence Between TPS Genes and Their Identifiers in *Artemisia annua*.

| Gene ID | Gene symbol | Gene ID | Gene symbol | Gene ID | Gene symbol |
| --- | --- | --- | --- | --- | --- |
| *chr1g00005461* | *AaTPS1* | *chr2g00342471* | *AaTPS22* | *chr6g00998471* | *AaTPS43* |
| *chr1g00057201* | *AaTPS2* | *chr2g00396651* | *AaTPS23* | *chr6g01036951* | *AaTPS44* |
| *chr1g00057441* | *AaTPS3* | *chr3g00542941* | *AaTPS24* | *chr6g01047441* | *AaTPS45* |
| *chr1g00057491* | *AaTPS4* | *chr4g00641521* | *AaTPS25* | *chr6g01047511* | *AaTPS46* |
| *chr1g00063911* | *AaTPS5* | *chr4g00670261* | *AaTPS26* | *chr8g01276451* | *AaTPS47* |
| *chr1g00063951* | *AaTPS6* | *chr4g00672871* | *AaTPS27* | *chr8g01286211* | *AaTPS48* |
| *chr1g00063961* | *AaTPS7* | *chr4g00672891* | *AaTPS28* | *chr8g01288801* | *AaTPS49* |
| *chr1g00065551* | *AaTPS8* | *chr4g00672901* | *AaTPS29* | *chr8g01309931* | *AaTPS50* |
| *chr1g00065601* | *AaTPS9* | *chr4g00700861* | *AaTPS30* | *chr8g01309941* | *AaTPS51* |
| *chr1g00065731* | *AaTPS10* | *chr4g00720511* | *AaTPS31* | *chr8g01337271* | *AaTPS52* |
| *chr1g00086741* | *AaTPS11* | *chr4g00728631* | *AaTPS32* | *chr9g01371181* | *AaTPS53* |
| *chr1g00120421* | *AaTPS12* | *chr5g00875231* | *AaTPS33* | *chr9g01371881* | *AaTPS54* |
| *chr1g00157351* | *AaTPS13* | *chr5g00885601* | *AaTPS34* | *chr9g01413051* | *AaTPS55* |
| *chr1g00168431* | *AaTPS14* | *chr5g00892201* | *AaTPS35* | *p0ctg_686g01637111* | *AaTPS56* |
| *chr1g00214731* | *AaTPS15* | *chr6g00969421* | *AaTPS36* | *Super-Scaffold_100155g01475221* | *AaTPS57* |
| *chr2g00242601* | *AaTPS16* | *chr6g00997791* | *AaTPS37* | *unctg_2583g01587621* | *AaTPS58* |
| *chr2g00266341* | *AaTPS17* | *chr6g00997821* | *AaTPS38* | *unctg_5787g01523991* | *AaTPS59* |
| *chr2g00266391* | *AaTPS18* | *chr6g00997851* | *AaTPS39* | *unctg_6058g01481911* | *AaTPS60* |
| *chr2g00290331* | *AaTPS19* | *chr6g00997881* | *AaTPS40* | *unctg_3130g01636731* | *AaTPS61* |
| *chr2g00330781* | *AaTPS20* | *chr6g00997901* | *AaTPS41* | *unctg_3674g01639751* | *AaTPS62* |
| *chr2g00330811* | *AaTPS21* | *chr6g00998171* | *AaTPS42* | *unctg_2482g01659011* | *AaTPS63* |

Supplementary Table S7 Correspondence Between TPS Genes and Their Identifiers in *A. lancea.*

| Gene ID | Gene symbol | Gene ID | Gene symbol | Gene ID | Gene symbol |
| --- | --- | --- | --- | --- | --- |
| *AtL_chr01G3636* | *AlTPS1* | *AtL_chr02G2120* | *AlTPS28* | *AtL_chr09G0387* | *AlTPS50* |
| *AtL_chr01G3640* | *AlTPS3* | *AtL_chr02G2143* | *AlTPS29* | *AtL_chr09G0390* | *AlTPS51* |
| *AtL_chr01G3642* | *AlTPS5* | *AtL_chr03G0558* | *AlTPS30* | *AtL_chr09G0396* | *AlTPS52* |
| *AtL_chr01G3643* | *AlTPS6* | *AtL_chr04G3822* | *AlTPS31* | *AtL_chr09G2189* | *AlTPS53* |
| *AtL_chr01G3730* | *AlTPS10* | *AtL_chr05G0473* | *AlTPS32* | *AtL_chr10G0098* | *AlTPS54* |
| *AtL_chr01G3734* | *AlTPS12* | *AtL_chr05G0475* | *AlTPS34* | *AtL_chr10G0133* | *AlTPS55* |
| *AtL_chr01G4212* | *AlTPS13* | *AtL_chr05G0477* | *AlTPS35* | *AtL_chr10G0251* | *AlTPS56* |
| *AtL_chr01G4217* | *AlTPS14* | *AtL_chr05G0478* | *AlTPS36* | *AtL_chr10G0254* | *AlTPS57* |
| *AtL_chr01G4230* | *AlTPS16* | *AtL_chr05G0479* | *AlTPS37* | *AtL_chr10G0260* | *AlTPS59* |
| *AtL_chr01G6342* | *AlTPS17* | *AtL_chr05G0488* | *AlTPS39* | *AtL_chr10G0491* | *AlTPS63* |
| *AtL_chr01G6344* | *AlTPS18* | *AtL_chr05G0492* | *AlTPS40* | *AtL_chr10G0500* | *AlTPS65* |
| *AtL_chr01G6351* | *AlTPS19* | *AtL_chr05G0497* | *AlTPS41* | *AtL_chr10G1209* | *AlTPS66* |
| *AtL_chr01G6355* | *AlTPS20* | *AtL_chr05G0499* | *AlTPS42* | *AtL_chr10G1225* | *AlTPS69* |
| *AtL_chr01G6403* | *AlTPS21* | *AtL_chr05G0502* | *AlTPS43* | *AtL_chr12G0378* | *AlTPS70* |
| *AtL_chr02G0473* | *AlTPS22* | *AtL_chr05G0510* | *AlTPS45* | *AtL_chr12G0379* | *AlTPS71* |
| *AtL_chr02G0475* | *AlTPS23* | *AtL_chr05G0512* | *AlTPS46* | *AtL_chr12G2850* | *AlTPS72* |
| *AtL_chr02G0476* | *AlTPS24* | *AtL_chr05G3849* | *AlTPS47* | *AtL_chr12G2858* | *AlTPS74* |
| *AtL_chr02G0518* | *AlTPS25* | *AtL_chr05G3881* | *AlTPS48* |  |  |
| *AtL_chr02G0869* | *AlTPS26* | *AtL_chr05G3901* | *AlTPS49* |  |  |


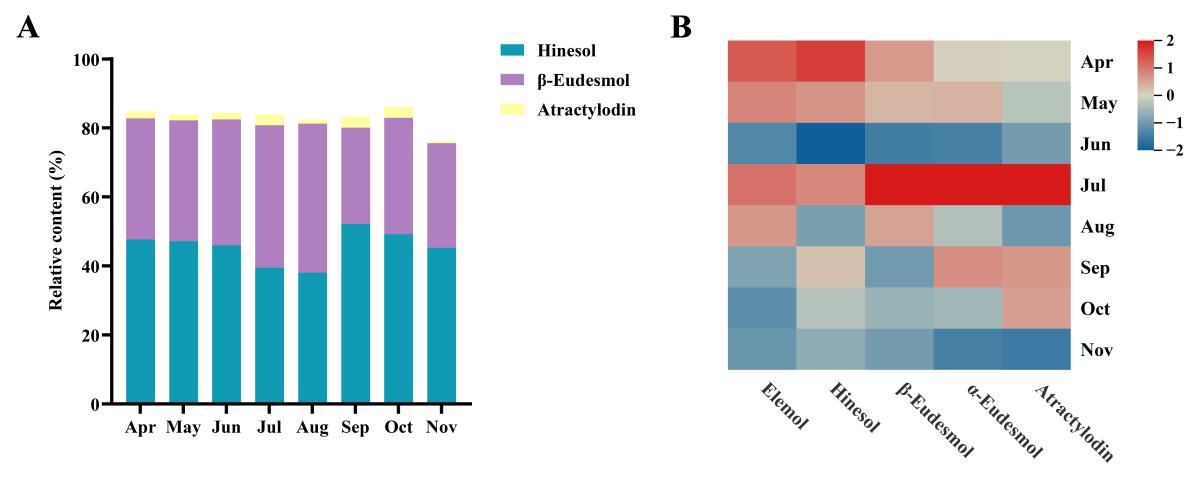


Supplementary Figure S1 **(A)** Total relative content of the three principal components, Hinesol, β-Eudesmol, and Atractylodin. **(B)** Heatmap analysis of five active components.


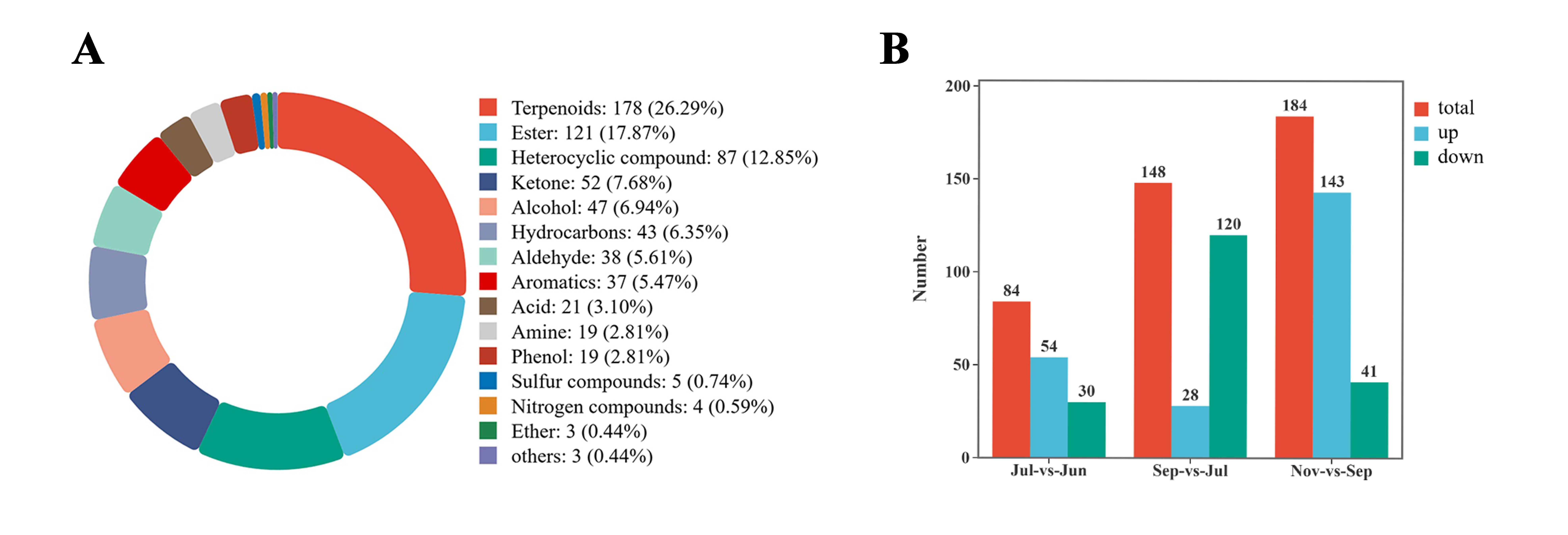


Supplementary Figure S2 **(A)** Classification of total metabolites across four developmental periods. **(B)** Differential metabolite analysis of three comparison groups: Jul-vs-Jun, Sep-vs-Jul, and Nov-vs-Sep.


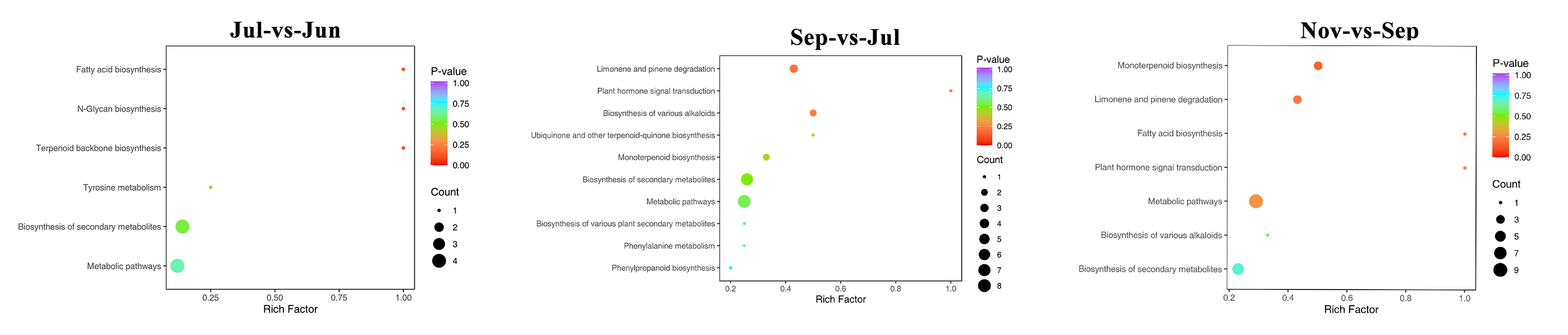


Supplementary Figure S3 KEGG enrichment analysis of differentially accumulated metabolites in the three comparison groups: Jul-vs-Jun, Sep-vs-Jul, and Nov-vs-Sep.


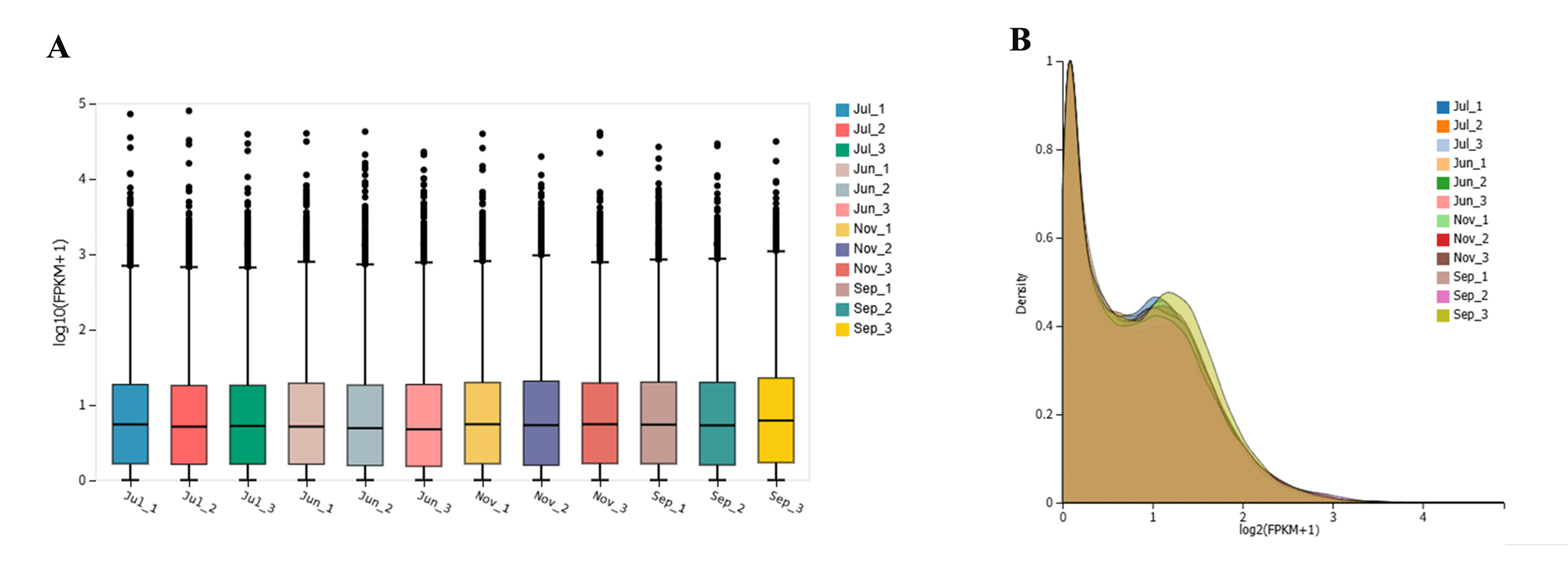


Supplementary Figure S4 **(A)** FPKM distribution boxplot. **(B)** Expression density distribution plot of the 12 samples.


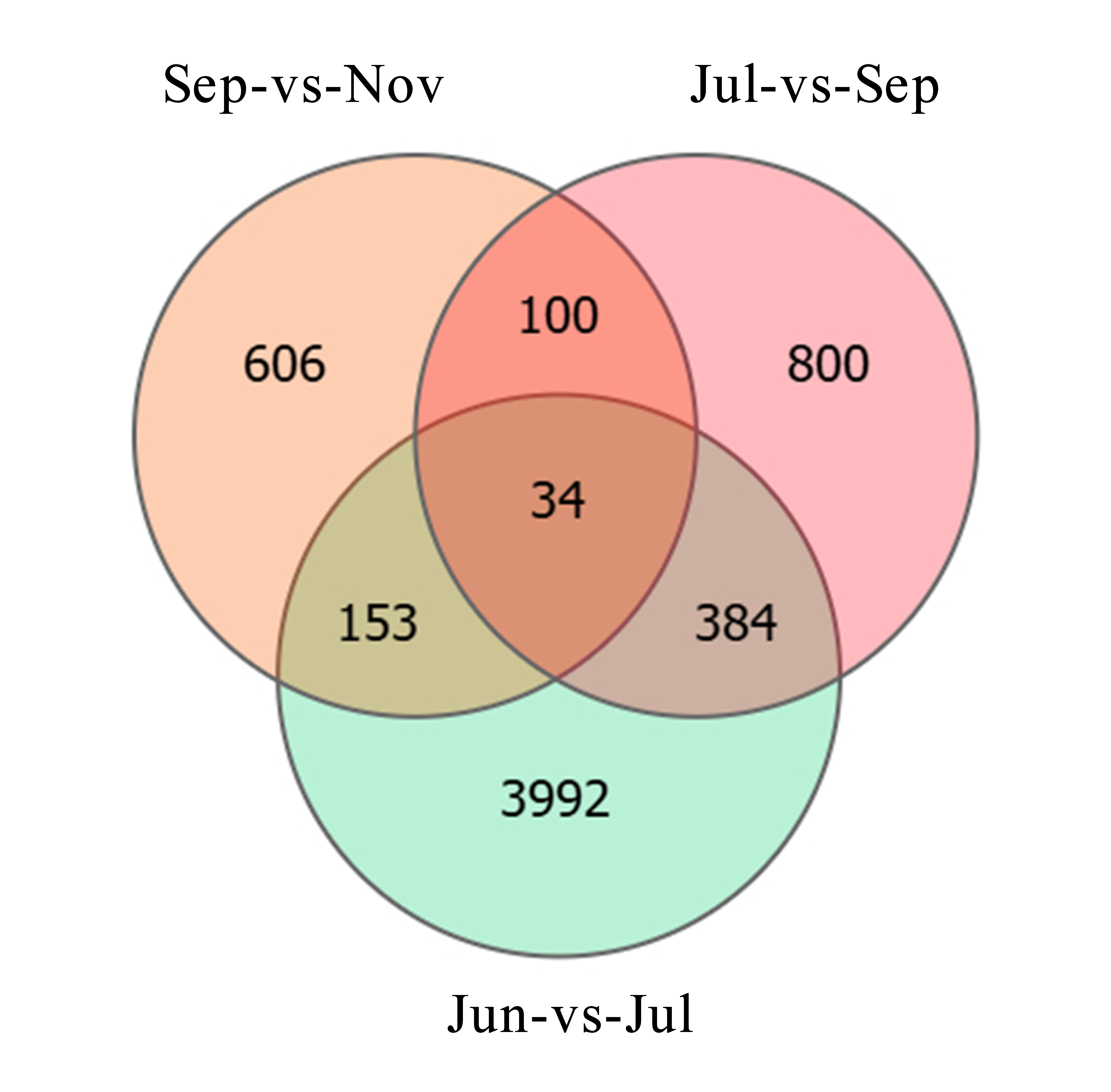


Supplementary Figure S3 Venn diagram of the three comparison groups: Jun-vs-Jul, Jul-vs-Sep, and Sep-vs-Nov.


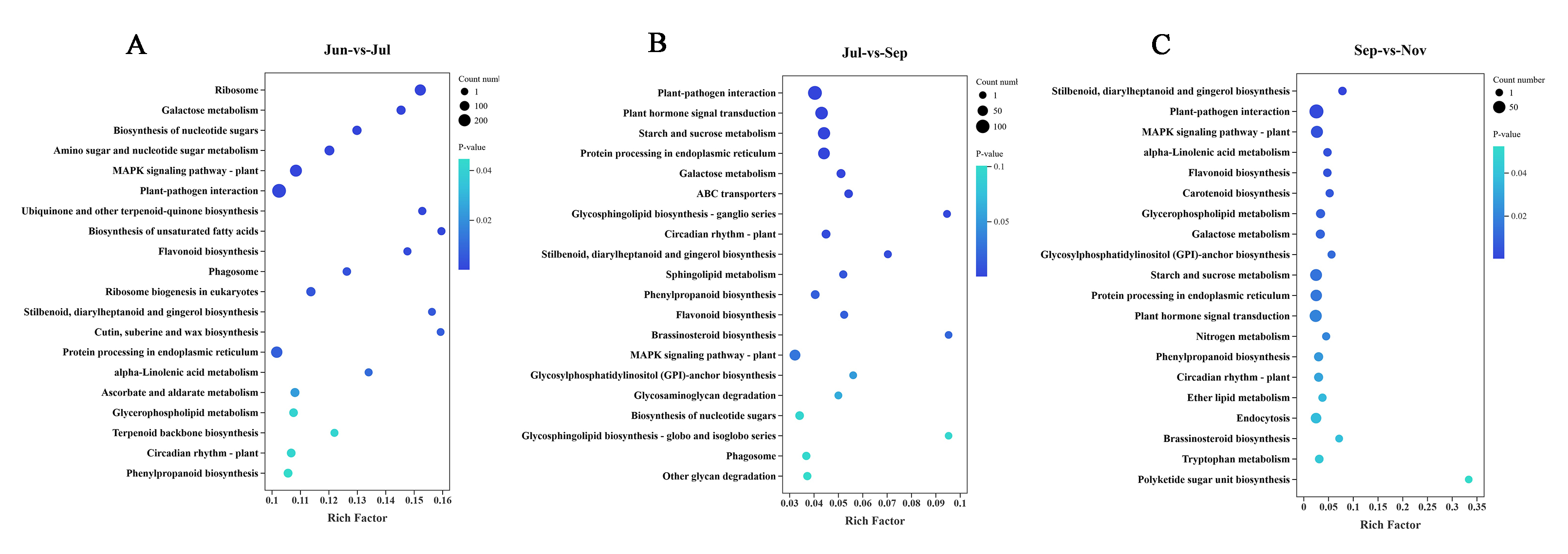


Supplementary Figure S6 KEGG enrichment analysis of differentially expressed genes (DEGs) in the three comparison groups: **(A)** Jun-vs-Jul. **(B)** Jul-vs-Sep. **(C)** Sep-vs-Nov.


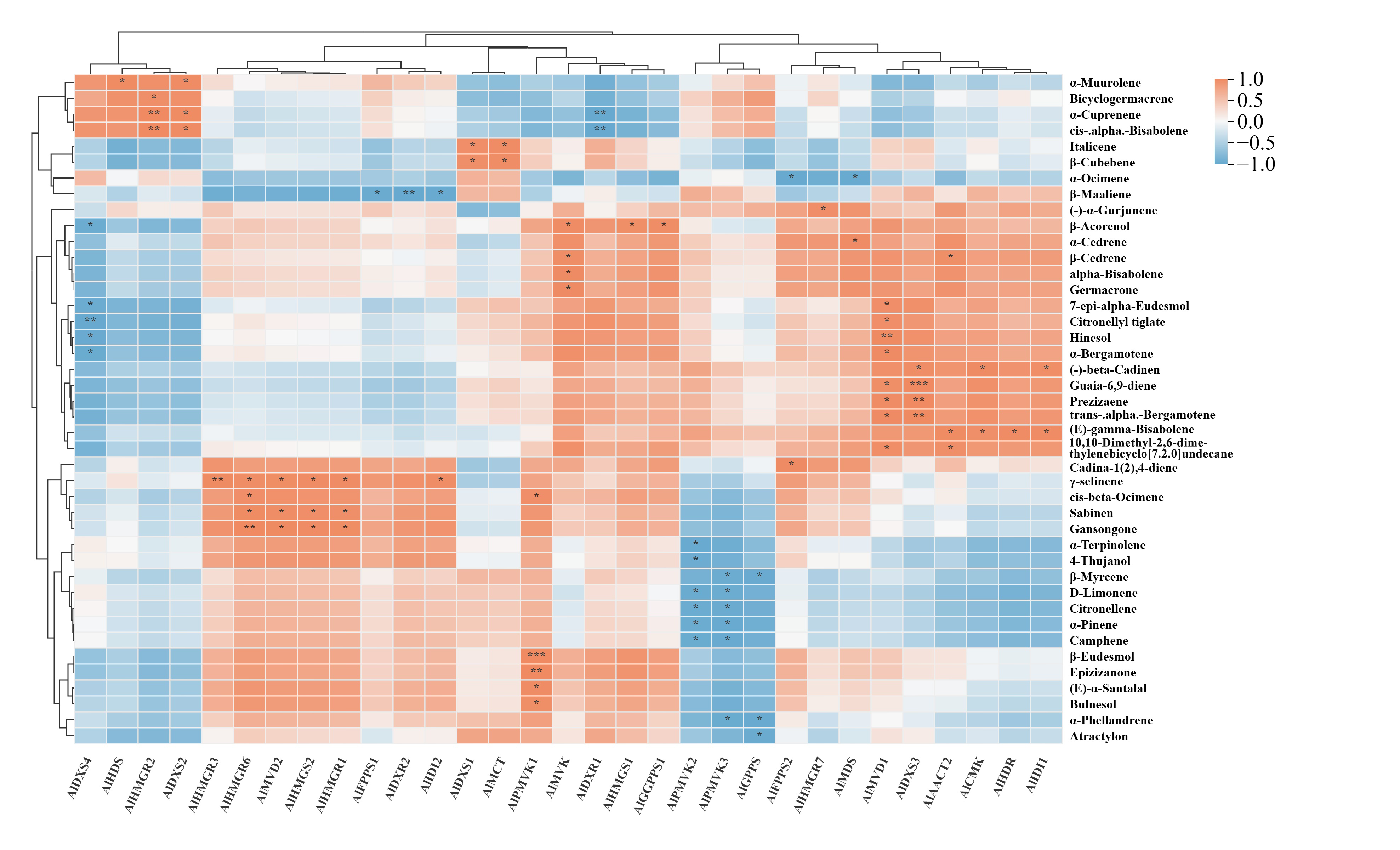


Supplementary Figure S7 Correlation between the top 50 most abundant terpenoid compounds and 36 upstream genes involved in the biosynthesis pathways. Orange indicates a positive correlation, while blue indicates a negative correlation. Significance levels are denoted by asterisks: * indicates 0.01 < p < 0.05, ** indicates 0.001 < p < 0.01, and *** indicates p ≤ 0.001.





Supplementary Figure S8 qRT-PCR results of six upstream genes involved in the terpenoid biosynthesis pathways.


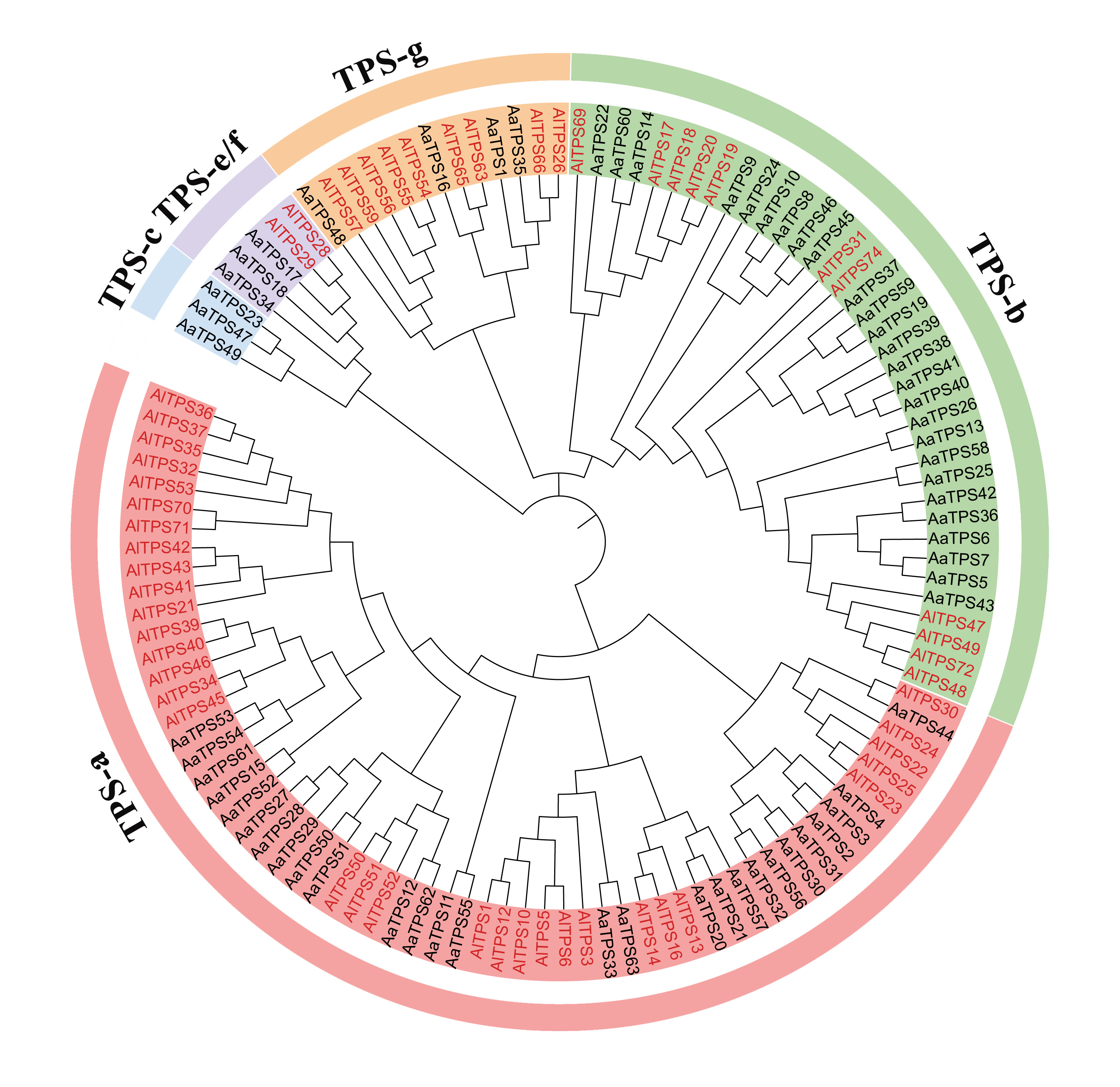


Supplementary Figure S9 Phylogenetic Analysis of Terpene Synthase Genes in *Artemisia annua* and *A. lancea*





Supplementary Figure S10 Conserved motifs of 12 terpenoid synthase genes.


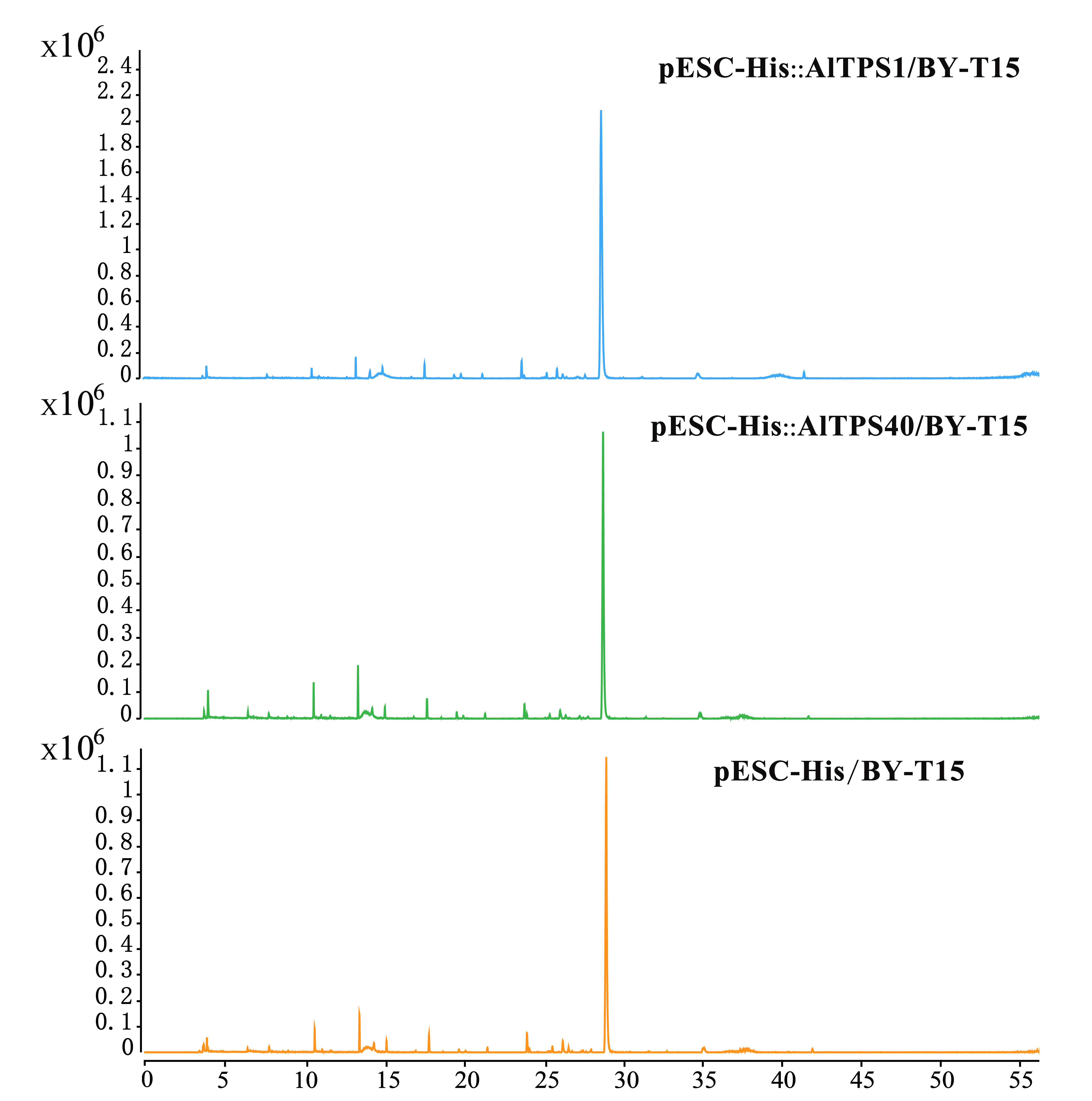


Supplementary Figure S11 The GC-MS total ion chromatogram of *AlTPS1* and *AlTPS40*.


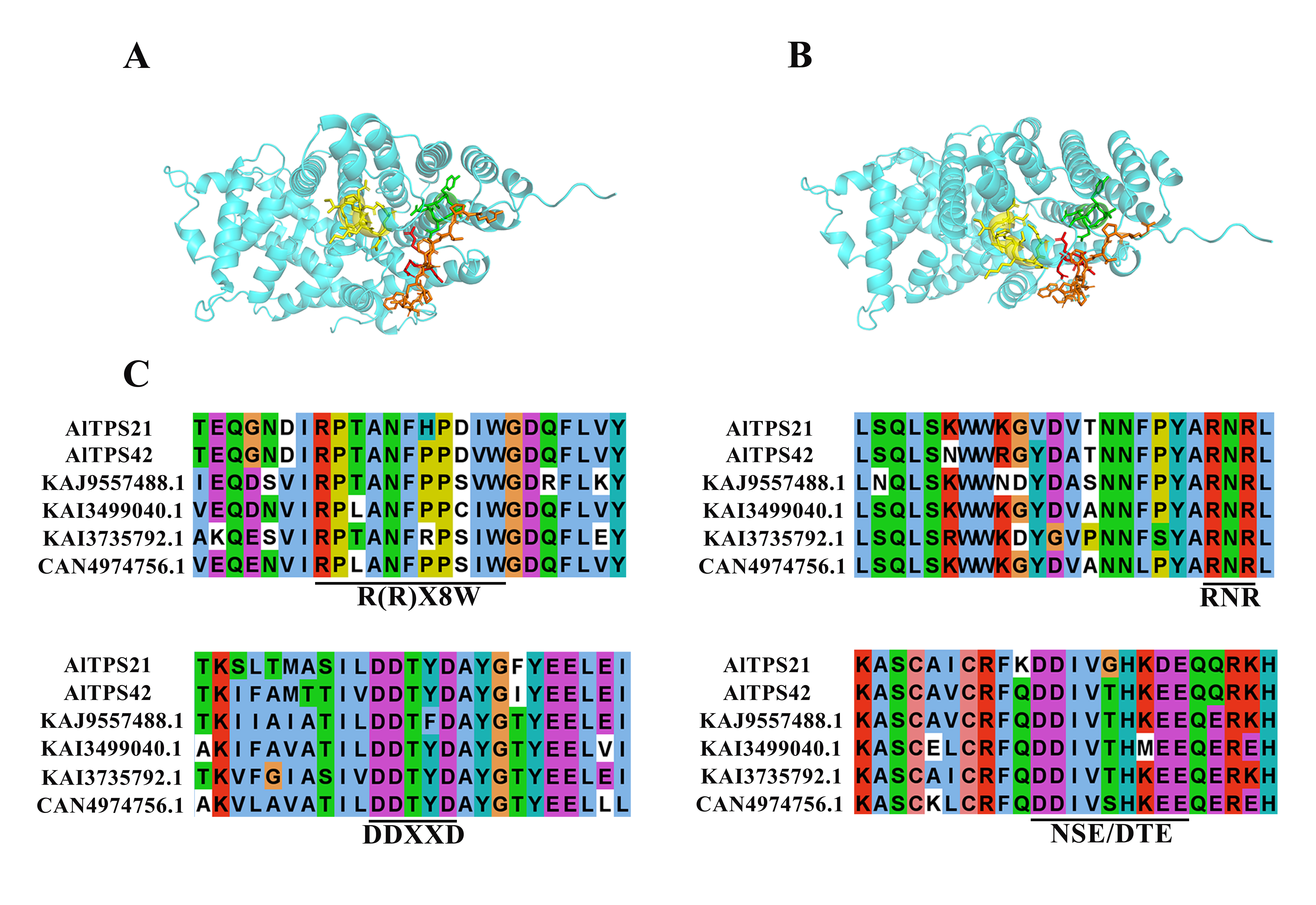


Supplementary Figure S12 Predicted protein structures of AlTPS21 and AlTPS42. **(A)** Tertiary structure of AlTPS21. **(B)** Tertiary structure of AlTPS42. **(C)** Conserved motifs of AlTPS proteins.
